# Integrative topological analysis of mass spectrometry data reveals molecular features with clinical relevance in esophageal squamous cell carcinoma

**DOI:** 10.1038/srep21586

**Published:** 2016-02-22

**Authors:** She-Gan Gao, Rui-Min Liu, Yun-Gang Zhao, Pei Wang, Douglas G. Ward, Guang-Chao Wang, Xiang-Qian Guo, Juan Gu, Wan-Bin Niu, Tian Zhang, Ashley Martin, Zhi-Peng Guo, Xiao-Shan Feng, Yi-Jun Qi, Yuan-Fang Ma

**Affiliations:** 1 Henan Key Laboratory of Cancer Epigenetics, Cancer Institute, The First Affiliated Hospital, College of Clinical Medicine, Henan University of Science and Technology, Luoyang, P. R. China, 471003; 2 Henan Key Laboratory of Engineering Antibody Medicine, Henan International United Laboratory of Antibody Medicine, Key Laboratory of Cellular and Molecular Immunology, Henan University School of Medicine, Kaifeng 475004, P.R. China; 3 School of Mathematics and Statistics, Henan University, Kaifeng, China, Henan 475004, P. R. China; 4 School of Cancer Sciences, College of Medical and Dental Sciences, University of Birmingham, Birmingham, UK

## Abstract

Combining MS-based proteomic data with network and topological features of such network would identify more clinically relevant molecules and meaningfully expand the repertoire of proteins derived from MS analysis. The integrative topological indexes representing 95.96% information of seven individual topological measures of node proteins were calculated within a protein-protein interaction (PPI) network, built using 244 differentially expressed proteins (DEPs) identified by iTRAQ 2D-LC-MS/MS. Compared with DEPs, differentially expressed genes (DEGs) and comprehensive features (CFs), structurally dominant nodes (SDNs) based on integrative topological index distribution produced comparable classification performance in three different clinical settings using five independent gene expression data sets. The signature molecules of SDN-based classifier for distinction of early from late clinical TNM stages were enriched in biological traits of protein synthesis, intracellular localization and ribosome biogenesis, which suggests that ribosome biogenesis represents a promising therapeutic target for treating ESCC. In addition, ITGB1 expression selected exclusively by integrative topological measures correlated with clinical stages and prognosis, which was further validated with two independent cohorts of ESCC samples. Thus the integrative topological analysis of PPI networks proposed in this study provides an alternative approach to identify potential biomarkers and therapeutic targets from MS/MS data with functional insights in ESCC.

Rapid advances in proteomics allow hundreds to thousands of molecular changes being simultaneously identified during progression of disease, providing a comprehensive picture of malfunction relative to healthy state^1^,^2^. Although fold change analysis together with standard statistical measure if sufficient number of replicates available is the most commonly used approach for the identification of potential biomarkers, the inherent constraints of this approach generally generate differentially expressed molecules with possibly high rates of false positives for low-abundance and of false negatives for high-abundance molecules, respectively^3^,^4^,^5^,^6^. More importantly, differentially expressed molecules extracted from various independent studies suffering low consistency pose difficulties in subsequent clinical application^7^,^8^,^9^,^10^. In addition, this approach can overlook biologically meaningful molecules without largest fold change such as transcription factors^4^. Furthermore, these aberrant changes lack the ability to link the functional importance with pathogenesis^11^ and pose challenges in interpretation from a biological and systemic perspective.

On the other hand, mass spectrometry (MS)-based proteomics currently widely used for biomarker discovery has incomplete proteome coverage of individual samples (limited fraction of proteins identified) and poor consistency across samples^11^,^12^. As genes known to be associated with the same phenotype tend to cluster together in protein-protein interaction (PPI) networks ascribing to sharing similar functions^13^,^14^,^15^,^16^,^17^,^18^, network-based methods can alleviate incomplete data coverage and inconsistency as well as complement cluster obtained via fold change analysis^11^,^19^. Moreover, network-based approaches have been extensively used for prioritization of drug target^20^ and identification of multiple disease markers, including breast cancer^7^,^21^,^22^,^23^, colon cancer^9^,^24^,^25^, prostate cancer^26^, ovarian cancer^16^, gastric cancer^27^, inflammatory response^28^,^29^, etc. Analysis of topological features of network, e.g. degree^30^,^31^, betweenness^32^,^33^, k-shell^34^, motif centrality^35^,^36^, has been a topic of great interest and been utilized to define critical points representing essentiality in biological networks and disease biomarkers as well^27^,^37^. Compared with differential expressions of individual proteins, network topology of proteins is more conserved across datasets and has the ability to provide otherwise information^37^. Therefore, combining MS-based proteomic data with network and hence topological features of such network could identify more clinically relevant molecules and meaningfully expand the repertoire of proteins returned via MS analysis.

Esophageal squamous cell carcinoma (ESCC) remains the predominant histological subtype of esophageal carcinoma (EC)^38^ and ranks as the fourth in terms of both incidence and mortality in China^39^. Long-term survival of advanced ESCC after surgery is dismal with a 5-year survival rate <25%, mainly due to late diagnosis, aggressive nature and limited treatment options^40^. Obviously, it is pressing to identify appropriate biomarkers for early diagnosis and therapeutic targets as well.

Here we used Isobaric Tags for Relative and Absolute Quantification (iTRAQ) combined with 2D-LC-MS/MS to globally identify differentially expressed proteins (DEPs) implicated in ESCC. To alleviate the weaknesses of MS-based proteomics, a PPI network was created by mapping 244 DEPs as seeds to a web-based PPI database. We identified structurally dominant nodes (SDNs) by integrative topological analysis of seven individual measures as potential molecular signatures for ESCC and determined the clinical relevance of these SDNs in comparison with DEPs and differentially expressed genes (DEGs) as well.

## Results

### Construction of protein-protein interaction network by DEPs in ESCC

Protein pools of ESCC and corresponding non-tumor epithelial tissue (N) after iTRAQ-labeling were MS/MS quantified. Using a threshold of 1.5-fold mean difference and two unique peptides for each protein, a total of 244 DEPs including 119 up-regulated and 125 down-regulated proteins, respectively, were identified (Supplementary Table S1).

**Figure 1:**
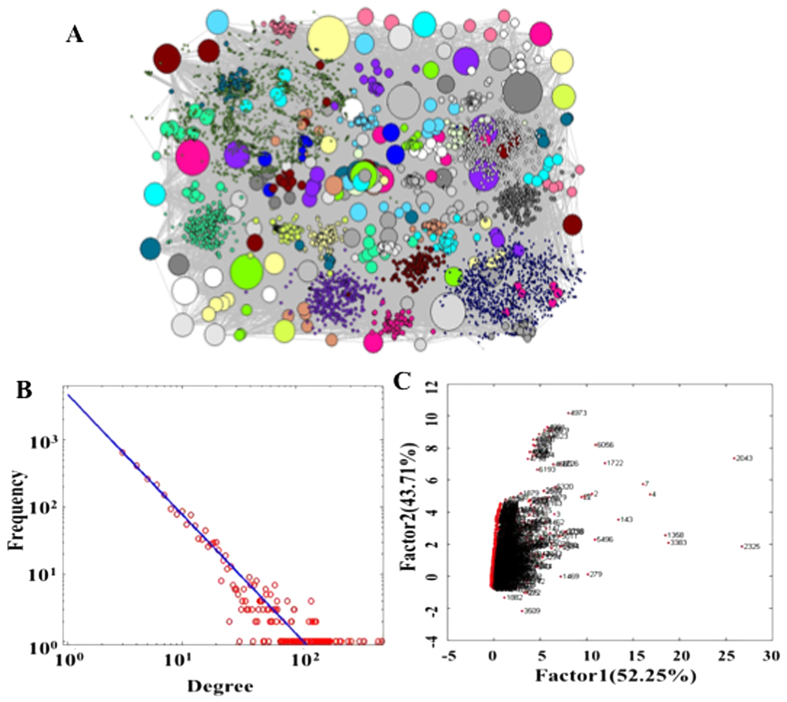
A PPI network construction by mapping 244 DEPs to a web-based HAPPI database and its topological features. (**A**) The seeds of 244 DEPs were mapped onto HAPPI database and were expanded to their first-degree neighbors, resulting in an extended network with 22 604 interactions between 6392 nodes. Different colors denote nodes with different degree and k-shell. (**B**) The degree distribution of the PPI network. (**C**) The contributions of the two factors in terms of factor scores 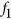
versus 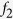
are 52.25% and 43.71%, respectively.

In the present study, the extended PPI network built by seeds of 244 DEPs resulted in 22 604 interactions between 6392 nodes (Fig. 1A). The statistical characteristics of the PPI networks are described in detail in Supplementary Table S2. The PPI network is sparse, with a connection density of 0.0011% and an average degree of 7.0726. Moreover, the degree distribution of the network is scale-free (Fig. 1B) and the power-law exponent is around −1.7770, which resembles another investigation on large-scale human PPI networks in reference^41^. Furthermore, the PPI network is small-world with very short average path length and high clustering coefficient, and the small-world SW index equals to 221.1198, which indicates the small-worldness of the network^41^.

### Identification of important nodes by integrative topological measures

A variety of topological measures have been proposed to assess the importance of nodes in complex networks from different perspectives. Resembling single molecular biomarkers, a single measure in PPI networks would not distinguish lethal proteins from the others. For in-depth identification of important nodes in PPI network implicated in ESCC, therefore, the present study integrated seven different topological measures, which comprised degree, betweenness, semi-local centrality, cluster coefficient, k-shell, PageRank and eigenvector centrality. After normalization, the seven topological measures were coalesced as two variables, i.e. principle component factor 1 and 2 (F1 and F2), which maintains 95.96% information of original seven topological measure (Fig. 1C). According to the values of F1 and F2, the top 50 nodes were selected as potential ESCC signature molecules named SDNs for further analysis.

### Concordance of differential protein and gene expression in ESCC

From five independent gene expression data sets, a total of 8 498 common genes present on all arrays were profiled on 186 ESCC patients partly including 87 pairs of ESCC and N, and exclusive 99 ESCCs of different clinical TNM stages (Table 1). To resemble a clinical practice, we used data set GSE 23400 to identify DEGs using two-sample T-test, a total of 1218 genes satisfying the *P* value < 0.0001 and q value (FDR) < 0.0001 were generated for further analysis (Supplementary Table S3).

Between 244 DEPs and 1218 DEGs, there were 67 common molecules detected by both proteomic and transcriptomic platforms from independent studies. Among the common molecules, 59 showed the same change direction including 22 up-regulated and 37 down-regulated molecules, respectively, while the other 8 showed opposite direction of deregulation (Table S4). Fisher’s exact test revealed that there was significant consistency between increased and decreased expression of 67 overlapping molecules (*P* = 1 × 10^−12^, Fisher’s exact test, Table S5).

### Clinical performance of SDN-based classifier compared with DEP-, DEG- and CF-based classifiers

For comparison, the top 50 of SDNs, DEPs and DEGs in terms of statistical *P* value were selected as potential signature molecules for building ESCC-related classifiers and the overlap between these molecules is rather small, i.e. four present (PPP2R1A, RPS15A, RPLP2 and RPSA) in SDNs and DEPs, one (RUVBL1) present in SDNs and DEGs, non-overlap between DEPs and DEGs (Table 2). Nevertheless, 23 molecules of 50 selected DEPs (46%) were virtually per se included in DEGs, 20 of 50 SDNs in DEPs (40%) and only 6 of SDNs in DEGs(12%) according to the cutoff value defined in our study.

Since the potential signature molecules for ESCC selected by various approaches might represent distinct aspects of tumor biology, comprehensive features (CFs) combining DEPs, DEGs and SDNs (a total of 150 potential molecules) would help us to build the most feasible classifier for clinical application. In the present study, the clinical relevance of SDN compared with DEP, DEG as well as CF was evaluated by classification performance in three different clinical settings, i.e. ESCC vs. N, early TNM stages (I–II) vs. late TNM stages (III–IV) and responder vs. non-responder to neoadjuvant chemoradiotherapy (neo-CRT).

**Figure 2:**
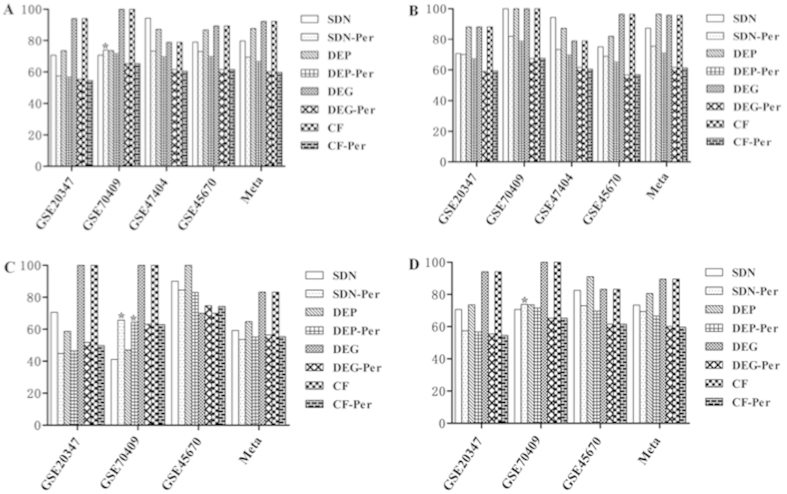
Clinical performances for discrimination of ESCC and N of SDN-, DEP-, DEG- and CF-based classifiers compared with 1000 random classifiers of each data set. (**A–D**) show the accuracies, sensitivities, specificities and AUCs of SDN-, DEP-, DEG- and CF-based classifiers compared with 1000 random classifiers of each data set for discrimination of ESCC and N, respectively. Note: * indicates higher values of permutated classifiers than SVM based classifiers in data set GSE 70409; SDN-, DEP-, DEG- and CF-Per indicate mean values of permutation tests.

Discrimination of ESCC and N By SVM analysis on the training set GSE 23400 including 53 pairs of ESCCs and adjacent Ns, LOO cross-validation was used to develop an optimal classifier. No significant differences in accuracies on training data set between the four different classifiers were observed (accuracies ranged from 91.5% to 94.3%). Furthermore, there were no significant differences as well with regards to accuracies, sensitivities, specificities and AUCs (*P* > 0.05, T-test) when the corresponding classifiers were performed on the four independent test cohorts and in meta-analysis (Fig. 2). It appears that SDN-based classifier tended to produce lower scores in most instances compared with the other three classifiers and the performance of CF-based classifier outperformed the others. The contributing molecules to optimal SDN signature (Table 3) were largest (9 molecules) followed by those of DEP-based classifier (7 molecules), while those of DEG- and CF-based classifiers were the same (3 molecules). Permutation test of 1000 random molecule sets indicated that all four classifiers generated in our study produced significantly better performance in meta analysis (*P* = 0.041 for SDN-, *P* < 0.001 for DEP-, DEG- and CF-based classifier, respectively). Figure 2A–D show the mean accuracies, sensitivities, specificities and AUCs of each test cohort and meta data sets from permutation tests. Except for four values for data set GSE 70409 marked by star in Fig. 2, the other values are superior to results of permutation test. However, all values of four classifiers generated in our study are higher than corresponding values of permutation test in meta analysis, suggesting that small sample size is the main contributing factor of inferior scores for certain individual test cohort.

**Figure 3:**
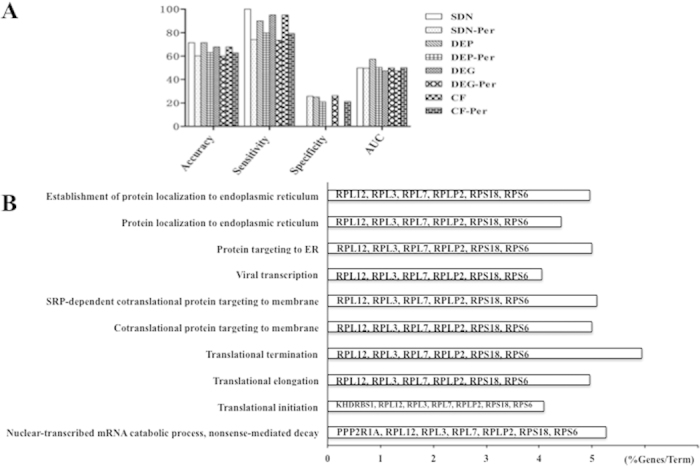
Clinical performance for discrimination of early from late TNM stages of SDN-, DEP-, DEG- and CF-based classifiers compared with 1000 random classifiers and enrichment of biological processes. (**A**) shows the accuracy, sensitivity, specificity and AUC of SDN-, DEP-, DEG- and CF-based classifiers and permutated classifiers for discrimination of early from late TNM stages. (**B**) shows the enriched biological processes for signature molecules of SDN-based classifier. Note: SDN-, DEP-, DEG- and CF-Per indicate mean values of permutation tests.

Discrimination of early vs. late TNM stages The data set GSE 23400 including 68 informative ESCCs was used as a training cohort and 28 ESCCs derived from data set GSE 45670 as a test cohort. In the training cohort, the performance of CF-based optimal classifier (75.0% accuracy) outstripped other classifiers with the minimal contributing molecules (8 molecules, Table 3)). In the test cohort of data set GSE 45670, the accuracy of SDN-based classifier increased from 64.7% to 71.4% while the performances of the other three classifiers decreased with the largest decrease (from 75.0% to 67.9%), moderate decrease (from 72.1% to 67.9%) and slight decrease (from 73.5% to 71.4%) in CF-, DEG- and DEP-based classifier, respectively (Fig. 3). The details of sensitivities, specificities and AUCs are shown in Fig. 3. The optimal DEG-based classifier consisting of 15 signature genes was the largest followed by those of SDN- and DEP-based classifiers (Table 3). Likewise, permutation test demonstrated better performance of our four SVM-based classifiers in terms of accuracy and sensitivity in independent test cohort but not for specificity and AUC (Fig. 3A). The *P* values of permutation test were 0.1, 0.038, 0.007, and 0.364 for SDN-, DEP-, DEG- and CF-based classifiers, respectively.

Predication of neoadjuvant chemoradiotherapy response Only one data set GSE 45670 including 28 ESCC patients profiled the global gene expression before and after neoadjuvant chemoradiotherapy response (neo-CRT). Due to the limited sample size, we used five-fold cross validation to measure the performance of four types of classifiers. The CF-based classifier with the largest contributing molecules (12 molecules) reached the highest prediction accuracy (92.9%) followed by SDN-based classifier (82.1%) with the least components (5 molecules). The prediction accuracy of pathological response for DEP- and DEC-based classifier was the same (78.6%) with similar contributing molecules (Table 3). Permutation test demonstrated significantly better performance of four investigated classifiers in our study after SVM-based classifier built using 1000 corresponding random molecule sets (*P* < 0.001).

### Enrichment analysis of GO biological processes and KEGG pathways

To understand the biological implications of molecular classifiers derived from different approaches, the constituent molecules of each classifier were analyzed for enrichment of Gene Ontology (GO) biological processes and Kyoto Encyclopedia of Genes and Genomes (KEGG) pathways. Only the signature molecules of SDN-based classifier for discrimination of TNM stages (15 molecules) were enriched for ribosome KEGG pathway and 10 biological processes mainly responsible for mRNA processing, protein translation and protein localization to organelles (Supplementary Table S6). Figure 3 shows the contributing molecules of 10 enriched biological processes, which include six high prevalent molecules of RPL12, RPL3, RPL7, RPLP2, RPS18 and RPS6. Although the protein biogenesis occurring in ribosome is not cancer-specific, these biological functions are indirectly linked to apoptosis, DNA repair and oncogenesis. However, no functional convergence was observed for signature molecules of the other classifiers in other distinct clinical settings.

### Experimental validation of ITGB1 in ESCC with different clinical stages and prognosis

**Figure 4:**
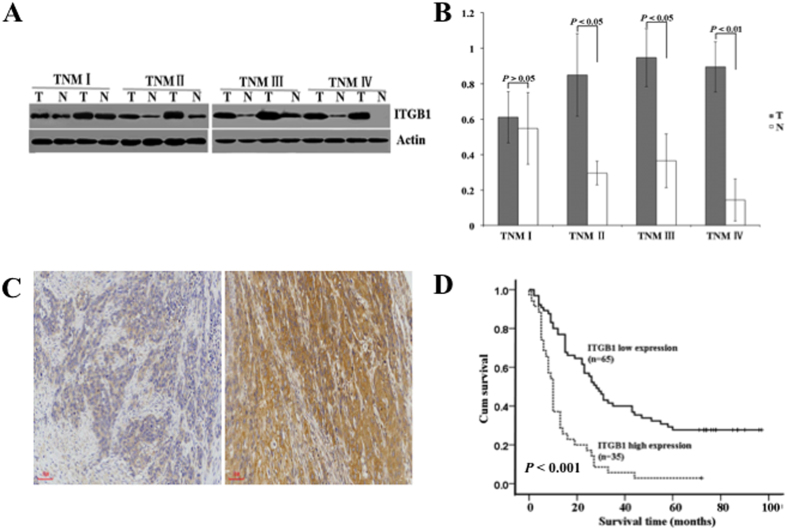
ITGB1 protein expression in ESCC and Kaplan-Meier survival curves of overall survival with regards to ITGB1 expression in ESCC. (**A**) Representative Western blots of ITGB1 protein expression level in ESCC and matched N with different TNM stages. (**B**) Quantification of ITGB1 protein expression in TNM I–IV ESCC and corresponding N. (**C**) Representative negative and positive immunoreactivity of ITGB1 in poorly-differentiated ESCC, respectively. (**D**) The 5-year overall survival curves of ESCC patients with low (n = 65) and high ITGB1 (n = 35) protein expression (*P* < 0.001).

The precise clinical staging assessment is essential for current management of EC and survival prediction although the current TNM staging system has critical limitations. Our results demonstrated that ITGB1 ranked 17th by integrative topological analysis and 3rd in the constituent molecules of SDN-based classifier for discrimination of early from late TNM stages. In addition, ITGB1 was not among the top 50 molecules of DEPs and DEGs for building the optimal SVM-based classifiers. Therefore, the clinical relevance of ITGB1 was evaluated by Western blot and immunohistochemistry (IHC) analyses in two independent cohorts of ESCC samples. With the progression of clinical stages, ITGB1 protein expression increased in a stepwise manner evidenced by Western blot and IHC (Fig. 4A–C). Furthermore, high expression of ITGB1 was correlated with late clinical stages in both cohorts (*P* = 0.019, *P* = 0.016, respectively) and with lymph node metastasis but with borderline significance (*P* = 0.057, *P* = 0.062, respectively). No significant correlations were observed between ITGB1 expression and other clinicopathological characteristics (Table 4). For cohort 1 with survival data after curative surgery, Kaplan-Meier survival analysis revealed that ESCC with high expression of ITGB1 had a significantly worse prognosis than ESCC with low expression (*P* < 0.001, Fig. 4D). The median survival time for ESCC patients with low expression of ITGB1 was 43.26 months whereas high expression of ITGB1 resulted in a remarkable shortened median survival time of about 13.86 months.

## Discussion

Apart from inherent limitations of fold-change and statistical measures to screen potential cancer biomarkers, long lists of differentially regulated molecules generated by high-throughput technologies fail to provide information at a biologically functional level^11^. Combining the MS/MS profiled data with biological networks had the ability to improve proteome coverage while unveil relationships between functionally related proteins^11^,^19^. In line with this viewpoint, the extended PPI network from 244 DEPs including 6392 nodes/molecules were far more than original 1567 proteins reported by iTRAQ MS/MS analysis. Topological features of biological networks are more conserved than differentially expressed molecules and provided more appropriate interesting molecules with discriminative potential^37^, suggesting that topological measures could identify distinct molecules of interest with clinical relevance.

Our results demonstrated that the integrative topological indexes comprising seven topological measures generated adequate clinical performance in three different clinical settings although there were few overlaps among the three sets of interesting molecules derived from different measures. In comparison with DEP- and DEG-based SVM classifier, the SDN-based classifier displayed more variation for discrimination ESCC from N on various transcriptomic profiling data sets with regards to accuracies, sensitivities, specificities and AUCs. For classification of clinical TNM stages, the performance of SDN-based classifier showed the largest change between the training cohort and the test cohort. The possible cause may lie in poor reproducibility of gene expression profiling and small sample size since SVM classifier used interesting molecules derived from DEGs between early and late TNM stages performed best in training cohort (88.2% accuracy) but worst in test cohort (25.0% accuracy, data now shown). For prediction of pathological response to neo-CRT, SDN-based SVM classifier produced the best accuracy compared with DEP- and DEG-based classifiers. Although the contributing molecules for each classifier did not overlap with the three molecules (MMP1, LIMCH1 and c1orf226) for constructing the predictive model of neo-CRT response^42^, our three models generated using interesting molecules from three different methods produced accuracies ranged from 78.5% to 82.1% and the CF-based SVM classifier produced higher accuracy (92.9%) than their original model (86% in training cohort and 81% in test cohort). Our results indicate that SDN- and CF-based classifier comprising biologically functional molecules performs better than other potential signature molecules selected only by statistical methods without functional relevance in more complex clinical settings like clinical TNM staging, treatment response and prognosis.

The overlap among the top 50 of DEGs and DEPs was only one, which poses severe concern with regards to clinical importance and application of these differentially expressed molecules. In addition, the distinct molecular profile unveiled by different approaches may depict parts of a panorama of tumor and integrative indexes derived from both platforms would improve our understanding of tumor biology and the clinical performance of these individual molecules. Therefore, biomarkers comprising multiple genes identified by different algorithms, which represent a complexity of multiple functional dysregulation, would provide more insightful understanding of malignant disease biology and consistently outperform individual genes across different populations^5^,^7^,^8^,^9^,^10^,^16^,^22^,^23^,^24^,^25^,^28^,^29^,^43^,^44^. Since SDN defined in the present study was an integrated index of seven topological measures of a human PPI network, the nodes with large absolute SDN values can well reflect their overall importance in the PPI network. Moreover, our previous investigations on the topological features of some functional genes in human PPI networks demonstrated that the functional genes were actually hallmark topological features^45^. Our study indicated that topological measures and differentially regulated molecules reflect distinct and complementary features of ESCC biology, and more importantly, integrative indexes of distinct features from various platforms or measures would produce the best performance, if not all, in certain clinical settings, by SVM analysis of total individual molecules.

Functional enrichment analysis provided biological explanations for the clinical performance of SDN-based TNM classifier. The contributing molecules of SDN-based TNM classifier were enriched in the pathway of ribosome and the biological processes in protein synthesis and intracellular localization. Ribosomes present in all living cells are cellular organelles for protein synthesis and comprise equal amounts of ribosomal proteins and rRNA in eukaryotic ribosomes. Ribosomal proteins maintain the balance of protein and RNA of itself and aberration in ribosome synthesis could lead to cell cycle arrest or to apoptosis. Cai *et al*. reported that reduced ribosomal biogenesis caused by RUNX1 resulted in a low metabolic profile and slow cell cycling, which provided a competitive advantage to pre-leukemic stem cells through increased stress resistance^46^. Dysregulation of ribosomal protein expression was responsible for cisplatin resistance in malignant cells of Hela^47^, EC109^48^ and EC9706^49^. Therefore, ribosomal proteins represent potential therapeutic targets evidenced by anti-tumor activities exerting by ribosome-inactivating proteins across various cancers^50^. In sharp contrast, the signature molecules of the other classifiers did not show any enriched biological features. Unlike SDN-based TNM signature molecules identified by network topological analysis, the constituent molecules of other clinical classifiers represent a combination of individual molecules without inherent functional linkage, which possess a variety of distinct molecular functions and preclude from identification of common biological themes. However, functional and pathway enrichment analysis of total DEPs and DEGs revealed biological traits more closely linked to cancer, such as p53 signaling pathway, cell cycle, keratinocyte differentiation, focal adhesion, adherens junction, pancreatic cancer, endometrial cancer, acute myeloid leukemia, etc. Nevertheless, the top 50 SDN molecules displayed the maximal enriched terms in terms of GO biological processes and KEGG pathways followed by DEGs, and DEPs were only enriched in biological processes of peptide cross-linking and epidermal cell differentiation (Supplementary Table S6).

As TNM staging provides useful information that helps predict the prognosis of cancer patients as well as tailor therapeutic interventions, we selected ITGB1, one contributing molecules to SDN-based TNM classifier, otherwise missed by differential measures for potential biomarkers, to validate its clinical stage and prognostic relevance. Both Western blot and IHC results demonstrated upregulation of ITGB1 protein expression correlated significantly with late TNM stages, which supports that topological analysis of network is a useful approach to identify potential biomarkers. Integrins mediated interactions and signaling of cell-cell and cell-extracellular matrix (ECM) are crucial for maintenance of tissue homeostasis, cell proliferation and survival^51^. Consistent with their multiple biological functions, altered expression or expression pattern of integrin correlates with tumor progression and prognosis. Increased expression of ITGB1 was observed in upper aerodigestive tract^52^, cervical SCC^53^ and vulval SCC^54^. Deletion of ITGB1 expression in VSCC cell line A431 or antagonizing ITBG1 antibody can inhibit the invasive ability both *in vitro* and *in vivo*^54^. A novel macrolide analog F806 suppressed more effectively ESCC cell growth *in vitro* and *in vivo* via initiation of anoikis and subsequent apoptosis by blocking ITGB1 activation compared with siRNA-mediated ITGB1 knockdown^55^. In contrast, other studies reported decreased expression in oral SCC^56^. Enhanced expression of ITGB1 at the tumor invasion front correlated with the absence of regional lymph node metastasis and the persistence of physiologically polarized expression of ITGB1 was significantly associated with favorable prognosis^57^. However, survival analysis of our ESCC patients revealed that increased ITGB1 expression was significantly associated with late TNM staging, worse prognosis and lymph node metastasis but with borderline significance. The discrepancy on biological function and clinical relevance of ITGB1 in different types of tumor may ascribe to variations of antibodies, ethnic origin, stage difference, tissue specificity, etc. which warrants further investigations to clarify.

## Conclusions

The present study demonstrates that integrative topological indexes derived from seven individual topological features carrying inherent functional linkage produce comparable classification performance in three different clinical settings. The signature molecules of SDN-based classifier for distinction of early from late clinical TNM stages were enriched in biological traits of protein synthesis, intracellular localization and ribosome biogenesis, which suggests that ribosome biogenesis represents a promising therapeutic target for treating ESCC. In addition, one of signature molecules of ITGB1 selected by topological measures correlated with clinical TNM stages and ESCC prognosis. Thus the integrative topological analysis of PPI networks proposed in this study provides an alternative approach to identify potential biomarkers and therapeutic targets from MS/MS data with functional insights in ESCC. By taking advantage of freely available human PPI networks, SDNs depending exclusively on the topological features would, to some extent, save costly and time-consuming laboratory experiments compared with other approaches for biomarker discovery.

## Methods

### Tissue samples

ESCC tissue samples for proteomic quantification were obtained from Linzhou Cancer Hospital, Henan, China, between 2010 and 2011. All patients gave informed consent before sample collection. None of ESCC patients received radio- or chemotherapy before surgery. This study was approved by the Ethnics Committee of the Medical School, Henan University, China and all methods in this study were carried out in accordance with the approved guidelines.

### Tissue sample preparation

Tissue samples were minced and homogenized on ice in lysis buffer containing 8 M urea, 4% CHAPS, 40 mM DTT and complete proteinase inhibitor cocktail (Roche). The tissue homogenates were centrifuged at 13.2 × 1000 rpm at 4 °C for 15 min to remove any insoluble debris and the supernatant was stored at −80 °C until use.

### iTRAQ labeling after protein trypsinization

Protein pools of ESCC and matched N were made by mixing of equal quantities of individual proteins from 10 ESCC and N, respectively, and then were precipitated by −20 °C acetone followed by resuspension. The dissolved protein was reduced, alkylated and subjected to trypsinization. The tryptic peptides of ESCC and N were pooled after iTRAQ labeling, and desalted by C18 SepPak column and dried in SpeedVac until complete dryness.

### MALDI-TOF/TOF Analysis

The labeled peptides were separated into 12 fractions by mixed-mode anion exchange/reverse-phase chromatography using a 2.1 × 150 mm Acclaim Mixed-mode WAX-1 HPLC column (Dionex, Camberley, UK) and a gradient of 0–40% B over 40 minutes (A: 20 mM ammonium formate pH 6.5, 3% acetonitrile, B: 2 mM ammonium formate pH 3.0, 80% acetonitrile) at a flow rate of 250 μL/min. Each fraction was dried, dissolved in 0.1% TFA and the peptides fractionated onto a 384 spot x 800 μm anchorchip using a 75 μm x 25 cm Acclaim PepMap 100 C18 HPLC column (Dionex, Camberley, UK), a 0–40% acetonitrile gradient in 0.1% TFA at 300 nl/min with in-line addition of matrix (5 mg/ml α-Cyano-4-hydroxycinnamic acid in 90% acetonitrile, 0.1% TFA, 1 mM NH4H2PO4) using a Proteineer fc II spoting robot (BrukerDaltonics, Bremen, Germany). Spectra were nearest-neighbour calibrated using Peptide Calibration Standard II (BrukerDaltonics, Bremen, Germany). Automated data acquisition was performed using a BrukerUltraflextreme MALDI-TOF/TOF instrument controlled via Warp-LC software (BrukerDaltonics, Bremen, Germany). Data were searched using MASCOT via Proteinscape (BrukerDaltonics, Bremen, Germany) against the SwissProt human sequence database (and a randomised version thereof) using tolerances of 20 ppm on precursor ions, 0.7 Da on fragment ions and a minimum peptide Mowse score of 30. Only proteins identified by two or more unique peptides were accepted. These criteria generated zero hits in the decoy database.

### Differentially expressed proteins and protein-protein interaction network construction

For identification of DEPs, a minimum of 2 unique peptides and 1.5 fold-difference was used. The DEPs were utilized as seed proteins to build a PPI network. The seed proteins were mapped onto a web-based Human Annotated and Predicted Protein Interaction (HAPPI) database (http://bio.informatics.iupui.edu/HAPPI/)^58^. The seeds were expanded to their first-degree neighbors on a high confidence grade 5 to build an extended and high-quality PPI network. The PPI network was visualized using the Pajek software.

### Identification of important nodes by integrative analysis of seven topological features

We defined SDN based on seven topological indexes. The seven topological indexes included degree, semi-local centrality, betweenness, k-shell, PageRank, cluster coefficient and eigenvector centrality^34^,^45^,^59^,^60^. For convenience, we denote the seven index vectors for a network as 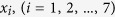.

To obtain the SDN, we used factorial analysis theory, which is a classical dimension reduction technology. This model describes variability among observed, correlated variables in terms of a potentially lower number of unobserved variables called factors. The observed variables can be modeled as linear combinations of the common factors and error terms. Generally speaking, the few common factors can reveal most of the information contained in the observed variables. Thus, the few common factors can be used later to reduce variables.

Suppose the 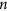 topological indexes for networks corresponding to a stochastic vector 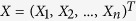, there are 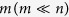 common factors 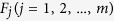 and 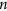 specific factors 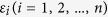. The index vectors 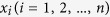 are realizations of 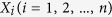. The orthogonal factor model can be established as:
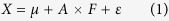
where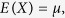 and
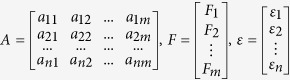


Based on the observation data 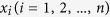, a key step of factorial analysis is to find the common factors 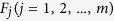 and then replace the original 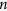 variables by 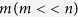 common factors 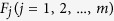.

The factorial model can be rotated to facilitate easier explanations of the common factors. The common factors 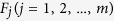 are actually linear combinations of the original variables.

For the network considered in this study, we find two common factors, which can reveal more than 95% information of the original seven topological indexes. The two common factors for the seven topological indexes of the PPI network are as follows:
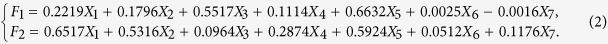


Based on the two common factors and the seven topological vectors of the PPI network, we derive the observation of 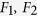 as factor scores 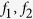. The values of the factor scores reflect the relative importance of each node. Factor scores 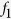 versus 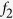 is shown in Fig. 1C. The contributions of the two factors are 52.25% and 43.71%, respectively. The overall contribution of the two factors can achieve as high as 95.96%, which indicate the two factors can reveal most of the information contained in the seven indexes. Further based on factor scores, we selected top 50 ranked nodes as shown in Table 2.

### Gene expression data sets

For clinical relevance evaluation, the present study adopted five publically available and independent gene expression data sets downloading from Gene Expression Omnibus (GEO) website (http://www.ncbi.nlm.nih.gov/gov, Table 1).

Each data set was acquired as CEL file and analyses were performed using BRB-ArrayTools. Probe sets missing greater than 20% in all readings in any single data set were removed from subsequent analyses. After normalization by reference array and combining multiple probe set into one per gene symbol, a total of 8 498 unique genes across five gene expression data sets were subsequently selected for further assessment of clinical relevance. DEGs were selected using a T-test with a q value threshold of 0.0001.

### Clinical relevance evaluation

In addition to DEPs-, DEGs- and SDN-derived potential signature molecules of ESCC, we surmised that combination of all above molecules of interest named comprehensive features (CFs) would help us to build the most feasible classifier for clinical application. To evaluate the clinical relevance of four different types of interesting molecules, we selected three different clinical settings. In clinical setting 1, classifiers were used to distinguish ESCC from adjacent N; in clinical setting 2, classification of early and late TNM stages was performed; in clinical setting 3, we used four different sets of molecules to predict the response to neo-CRT for ESCC.

To compare the clinical relevance of different types of interesting molecules, a radial basis functional support vector machine (SVM), which adopted a recursive feature elimination algorithm to select useful features, was used for building SDN-, DEP-, DEG- and CF-based classifier. The performance of SVM was estimated using five-fold cross validation error. Leave-one-out (LOO) cross validation was used to determine the optimal values of the kernel parameters and regularization parameter C, and the test error was obtained using the tuned parameters. The top 50 molecules according to statistical score were used as the feature vector for building the optimal classifier. Receiver operating characteristic (ROC) curves were plotted using sensitivity versus 1-specificity, and the areas under the curves (AUCs) were computed to evaluate the classification accuracies of three different classifiers with regards to ESCC and N, early and advanced TNM stages. Permutation test was used to compare the performances of optimal SVM-based classifiers with 1000 classifiers comprising molecule sets of the same size randomly selected from 8498 common genes present on all arrays.

### Functional enrichment analysis

A Cytoscape plug-in ClueGo that visualizes the selected terms in a functionally grouped network was used to estimate the biological relevance of each optimal classifier. The enrichment analyses of GO biological processes and KEGG pathways were performed using GO annotations for the complete human proteome as a reference set and the constituent molecules of each optimal classifier as a test dataset. The hyper-geometric test was used for enrichment analysis and the terms with a significance level of P < 0.0001 were regarded as over-represented after multiple testing correction method Benjamini and Hochberg for false discovery calculation.

### Western blot and immunohistochemistry

Western blot and IHC analyses of ITGB1 protein expression in ESCC were performed as previously described. The ESCC tissue microarray of cohort 1 (HEso-Squ180Sur-04) purchased from Shanghai Outdo Biotech Co., Ltd. comprised 100 ESCC patients undergoing surgery between 2006 and 2008. Cohort 2 included 91 ESCC patients undergoing esophagectomy surgery from 2010 to 2014 at the First Affiliated Hospital of Henan University of Science and Technology and Anyang people’s hospital. The composite immunostaining scores were calculated by multiplying the staining intensity and positivity.

### Statistics

All statistical analyses were performed using SPSS 16.0 software (SPSS, Chicago, IL, USA). Wilcoxon signed-rank test was used to evaluate the significance of the differences in ITGB1 expression normalized to β-actin. ROC curve analysis was used to determine the cutoff score of immunostaining. The chi-square test or Fisher’s exact test were used to evaluate the correlations between DEPs and DEGs, ITGB1 expression and clinicopathological features.

## Additional Information

**How to cite this article**: Liu, R.-M. *et al*. Integrative topological analysis of mass spectrometry data reveals molecular features with clinical relevance in esophageal squamous cell carcinoma. *Sci. Rep.* **6**, 21586; doi: 10.1038/srep21586 (2016).

## Supplementary Material

Supplementary Information

## Figures and Tables

**Table 1:** Characteristics of gene expression datasets.

**Dataset ID**	**No. of cases (N vs. ESCC)**	**Platform**	**Array type**
GSE23400	53 vs. 53	GPL96	[HG-U133A] Affymetrix Human Genome U133A Array
GSE20347	17 vs. 17	GPL571	[HG-U133A_2] Affymetrix Human Genome U133A 2.0 Array
GSE70409	17 vs. 17	GPL13287	Phalanx Human OneArray [Annotation HOA5 release 1.0]
GSE47404	0 vs. 71	GPL6480	Agilent-014850 Whole Human Genome Microarray 4 × 44 K G4112F (Probe Name version)
GSE45670	10 vs. 28	GPL570	[HG-U133_Plus_2] Affymetrix Human Genome U133 Plus 2.0 Array

**Table 2:** The top 50 molecules in order of statistical power for building ESCC-related classifiers.

**Approaches**	**Top 50 molecules**			
SDNs				
GRB2	FN1	MAPK1	CTNNB1	YWHAG
YWHAZ	ACTB	EEF1A1	UBC	STAT3
YWHAE	ALB	YWHAB	PPP2R1A	RPS3
ITGB	SYK	CAV1	STAT1	DDB1
EEF1G	RUVBL2	YBX1	YWHAH	RBM8A
**RUVBL1**	KPNB1	RPS6	FLNA	RPL3
HNRNPA1	RPS8	PSMD2	ACTN1	RPS15A
HNRNPU	SF3B3	KHDRBS1	HNRNPM	RPL12
RPL18	RPLP2	RPS17	RPS2	RPL7
RPS18	RPS28	EIF2S1	RPSA	RPL10
DEPs				
DDOST	HTATSF1	TFRC	RPS15A	KRT17
PPP2R1A	SH3BGRL	MCM4	RPLP2	VCAN
PKP3	AKR1A1	CYB5R3	PSMA5	LAMP2
S100A11	CCT5	RPSA	PSMC1	SERPINH1
ARL8B	CTSB	EFHD2	TAGLN2	NNMT
CRNN	CSTB	FLG	TGM3	SPINK5
RALY	SELENBP1	ZNF185	SPRR3	A2ML1
TGM1	CRABP2	IL1RN	AQP1	SPRR1A
MUC5B	MYLK	TPM2	GRHPR	AKAP12
CSTA	YAP1	TXN	SLC9A3R1	IVL
DEGs				
RFC4	CBX3	ECT2	COL1A1	MMP1
MFAP2	KIF4A	CKS1B	SPP1	MCM6
MCM2	PLAU	AGRN	BUB1B	KIF14
GINS1	BID	CDK1	NUP155	ATP2C1
CEP55	PDIA6	SNAI2	ACLY	ITPR3
PLXNA1	ACTL6A	FSCN1	RPN1	UBE2C
KIF2C	DLGAP5	SOX4	CENPF	PTK7
RANBP1	DNMT1	NUDT1	COL7A1	DTL
CDH11	FANCI	KIF20A	**RUVBL1**	ATR
MEST	FZD6	CENPA	EFNA1	CRYL1

Note: Underlined and bold molecules denotes overlaps between SDNs and DEPs, SDNs and DEGs, respectively.

**Table 3:** Signature molecules of SDN-, DEP-, DEG- and CF-based classifiers in three clinical settings.

**Classifier**	**Signature molecules**				
ESCC vs. N					
SDN-based	EIF2S1	YWHAH	RPLP2	RBM8A	PSMD2
	RPL3	YBX1	ACTN1	KHDRBS1	
DEP-based	RALY	CRNN	DDOST	CCT5	CRABP2
	RPLP2	CTSB			
DEG-based	CKS1B	COL1A1	CEP55		
CF-based	CKS1B	COL1A1	CEP55		
Early vs. late TNM stages					
SDN-based	RPL7	CAV1	ITGB1	RPS18	RPL3
	KHDRBS1	RPS6	STAT3	RPLP2	YBX1
	RPL12	RUVBL1	STAT1	PPP2R1A	SF3B3
DEP-based	HTATSF1	TGM3	AKAP12	IVL	CCT5
	RALY	CTSB	MUC5B		
	MCM2	FZD6	CBX3	AGRN	MCM6
DEG-based	COL1A1	FSCN1	BID	RANBP1	PDIA6
	MFAP2	ACLY	SNAI2	CDH11	EFNA1
	ATR				
CF-based	HTATSF1	TGM3	AKAP12	CBX3	PDIA6
	MCM6	MCM2	AGRN		
Responders vs. non-responders					
SDN-based	YBX1	EIF2S1	SF3B3	YWHAE	YWHAZ
DEP-based	DDOST	TXN	SPRR3	TPM2	SLC9A3R1
	CRNN	KRT17	CCT5	RPSA	PKP3
	TFRC				
DEG-based	MMP1	FANCI	PLAU	FSCN1	PTK7
	BID	KIF2C	CRYL1	GINS1	UBE2C
	YBX1	DDOST	FANCI	TPM2	PLAU
CF-based	TXN	SPRR3	PTK7	MMP1	TFRC
	EIF2S1	KIF2C			

**Table 4:**
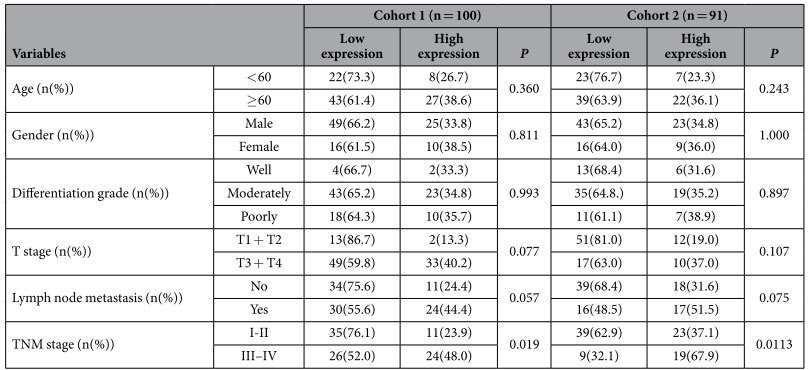
Association between ITGB1 expression and clinicopathological parameters of ESCC.
