# Supplementary material for: Integrative topological analysis of mass spectrometry data reveals molecular features with clinical relevance in esophageal squamous cell carcinoma
Source: Sci Rep. 2016 Feb 22;6:21586. doi: 10.1038/srep21586 (PMC4761933; doi:10.1038/srep21586)
Supplement: Supplementary Information [file srep21586-s1.pdf]

Integrative topological analysis of mass spectrometry data reveals molecular features with clinical relevance in esophageal squamous cell carcinoma

Rui-Min Liu[1,#], She-Gan Gao[2,#], Yun-Gang Zhao[1], Pei Wang[3], Douglas G. Ward[4], Guang-Chao Wang[1], Xiang-Qian Guo[1], Juan Gu[1], Wan-Bin Niu[1], Tian Zhang[1], Ashley Martin[4], Zhi-Peng Guo[1], Xiao-Shan Feng[2], Yuan-Fang Ma[1,*], Yi-Jun Qi[1,*]

1 Key Laboratory of Cellular and Molecular Immunology, College of Medicine, Henan University, Kaifeng, Henan 475004, P. R. China; 2 Department of Oncology, Cancer Institute, The first Affiliated Hospital of Henan University of Science and Technology, Luoyang 471003, P. R. China; 3 School of Mathematics and Statistics, Henan University, Kaifeng, China, Henan 475004, P. R. China; 4 School of Cancer Sciences, College of Medical and Dental Sciences, University of Birmingham, Birmingham, UK

[#]These authors contributed equally to this work.

*To whom correspondence should be addressed: Dr. Yi-Jun Qi or Prof. Yuan-Fang Ma, Key Laboratory of Cellular and Molecular Immunology, College of Medicine, Henan University, Kaifeng, Henan 475004, P. R. China. Tel: 86-371-23880398; Fax: 86-371-23880398; E-mail:qiyijun@hotmail.com or mayf@henu.edu.cn

Running title: Integrative topological analysis of MS/MS data of ESCC

Rui-Min Liu, lrmzzx@163.com;

She-Gan Gao, gsg112258@163.com;

Yun-Gang Zhao, 530713372@qq.com;

Pei Wang, wp0307@126.com;

Douglas G. Ward, D.G.WARD@bham.ac.uk;

Guang-Chao Wang, wgc119@foxmail.com;

Xiang-Qian Guo, baozidaoguo@163.com;

Juan Gu, 980807185@qq.com;

Wan-Bin Niu, 547566492@qq.com;

Tian Zhang, zhangtian90@126.com;

Ashley Martin, ashleymartinpost@gmail.com;

Zhi-Peng Guo, 875335218@qq.com;

Xiao-Shan Feng, samfeng137@hotmail.com

Table S1 DEPs identified by iTRAQ and tandem MS quantification.

Protein	Scores	RMS90 [ppm]	Peptides	Ratio (ESCC:T)
**Up-regulated proteins identified by iTRAQ and MS/MS quantification**				
Macrophage-capping protein	241.4 (M:241.4)	10.28	3	1.506
Tubulin alpha-4A chain	581.0 (M:581.0)	11.98	7	1.51
Actin-related protein 2	269.5 (M:269.5)	11.24	5	1.522
Annexin A7	225.5 (M:225.5)	2.82	4	1.522
Arginyl-tRNA synthetase, cytoplasmic	92.8 (M:92.8)	6.04	2	1.522
Catenin beta-1	174.1 (M:174.1)	4.11	3	1.522
Serine/threonine-protein phosphatase 6 catalytic subunit	112.5 (M:112.5)	22.14	2	1.522
Sulfide:quinone oxidoreductase, mitochondrial	130.4 (M:130.4)	3.25	2	1.522
Heterogeneous nuclear ribonucleoprotein A1	377.1 (M:377.1)	4.44	6	1.527
Proteasome activator complex subunit 1	170.0 (M:170.0)	12.10	3	1.527
26S proteasome non-ATPase regulatory subunit 2	166.5 (M:166.5)	11.21	2	1.531
Galectin-3-binding protein	79.6 (M:79.6)	11.68	2	1.539
Clusterin	167.7 (M:167.7)	9.89	2	1.543
Prolyl endopeptidase	216.4 (M:216.4)	7.88	2	1.547
Fibulin-1	776.8 (M:776.8)	7.28	7	1.555
Vacuolar protein sorting-associated protein 4B	89.0 (M:89.0)	11.11	2	1.555
26S protease regulatory subunit S10B	108.0 (M:108.0)	0.87	2	1.559
Collagen alpha-2(I) chain	281.7 (M:281.7)	11.20	5	1.563
Dolichyl-diphosphooligosaccharide--protein glycosyltran	243.2 (M:243.2)	8.46	3	1.567
Collagen alpha-3(VI) chain	350.6 (M:350.6)	7.85	5	1.571
Desmocollin-3	221.0 (M:221.0)	5.39	3	1.588
Signal transducer and activator of transcription 3	93.4 (M:93.4)	13.20	2	1.588
V-type proton ATPase catalytic subunit A	340.9 (M:340.9)	6.65	3	1.588
40S ribosomal protein S3	272.1 (M:272.1)	10.64	3	1.592
Reticulocalbin-3	262.9 (M:262.9)	11.03	4	1.592
Lumican	171.4 (M:171.4)	4.56	2	1.596
Voltage-dependent anion-selective channel protein 1	134.7 (M:134.7)	8.02	3	1.596
ATP-dependent DNA helicase 2 subunit 1	285.1 (M:285.1)	11.63	2	1.6
Vigilin	123.5 (M:123.5)	1.05	2	1.6
Profilin-1	353.4 (M:353.4)	4.58	5	1.604
NADPH--cytochrome P450 reductase	248.9 (M:248.9)	8.35	3	1.608
Alpha-actinin-1	1141.2 (M:1141.2)	2.37	11	1.616

Table S1 Proteomic profiling of ESCC by iTRAQ and tandem MS quantification

Protein	Scores	RMS90 [ppm]	Peptides	Ratio (ESCC:T)
Fibronectin	1223.4 (M:1223.4)	8.44	18	1.616
Aldo-keto reductase family 1 member C3 - Homo sapiens	203.3 (M:203.3)	9.68	2	1.62
Alanyl-tRNA synthetase, cytoplasmic	140.7 (M:140.7)	5.97	2	1.629
Actin-related protein 3	622.5 (M:622.5)	2.88	6	1.645
ADP/ATP translocase 3	136.0 (M:136.0)	6.48	3	1.645
Eukaryotic translation initiation factor 2 subunit 2-like pr	274.3 (M:274.3)	5.86	3	1.649
Ras-related protein Rab-2B	199.4 (M:199.4)	4.89	3	1.649
Ubiquitin carboxyl-terminal hydrolase 14	117.4 (M:117.4)	5.05	2	1.649
Procollagen-lysine,2-oxoglutarate 5-dioxygenase 3	115.5 (M:115.5)	9.53	2	1.665
Ras-related protein Rap-1A	235.5 (M:235.5)	2.94	3	1.665
Collagen alpha-1(I) chain	278.7 (M:278.7)	9.07	4	1.669
Nucleobindin-1	206.0 (M:206.0)	13.37	4	1.669
6-phosphofructokinase type C	216.8 (M:216.8)	7.82	3	1.673
Glucose-6-phosphate isomerase	165.0 (M:165.0)	1.74	2	1.673
T-complex protein 1 subunit beta	190.4 (M:190.4)	16.88	2	1.678
Vesicle-trafficking protein SEC22b	210.0 (M:210.0)	5.24	3	1.678
Annexin A5	800.5 (M:800.5)	3.92	7	1.686
Signal transducer and activator of transcription 1-alpha/b	134.6 (M:134.6)	3.32	2	1.69
Eukaryotic translation initiation factor 5A-1	166.9 (M:166.9)	21.67	2	1.71
Heterogeneous nuclear ribonucleoprotein A3	206.9 (M:206.9)	7.47	4	1.71
Plastin-3	252.2 (M:252.2)	5.00	4	1.714
Calnexin	670.3 (M:670.3)	3.44	10	1.722
Matrix-remodeling-associated protein 5	178.3 (M:178.3)	4.91	2	1.727
60 kDa SS-A/Ro ribonucleoprotein	151.2 (M:151.2)	9.53	2	1.731
Non-POU domain-containing octamer-binding protein	295.4 (M:295.4)	8.25	5	1.747
Periostin	553.5 (M:553.5)	3.68	6	1.747
Protein disulfide-isomerase A4	629.8 (M:629.8)	5.96	5	1.747
Basigin	76.5 (M:76.5)	6.03	2	1.755
Vacuolar protein sorting-associated protein 26A	73.2 (M:73.2)	8.72	2	1.763
Alpha-enolase	966.4 (M:966.4)	9.98	11	1.767
KH domain-containing, RNA-binding, signal transductio	186.3 (M:186.3)	21.17	2	1.767
Splicing factor, arginine/serine-rich 13A	114.3 (M:114.3)	7.11	2	1.767
Proteasome subunit alpha type-6	302.1 (M:302.1)	5.11	3	1.776
Membrane-associated progesterone receptor component 2	281.5 (M:281.5)	10.33	2	1.78

Table S1 DEPs identified by iTRAQ and tandem MS quantification.

Table S1 Proteomic profiling of ESCC by iTRAQ and tandem MS quantification

Protein	Scores	RMS90 [ppm]	Peptides	Ratio (ESCC:T)
Non-secretory ribonuclease	170.5 (M:170.5)	8.53	2	1.78
Aldo-keto reductase family 1 member C1 - Homo sapiens	343.6 (M:343.6)	5.51	4	1.784
Protein unc-45 homolog A	81.0 (M:81.0)	15.93	2	1.788
Legumain	202.3 (M:202.3)	14.01	2	1.804
40S ribosomal protein S2	134.5 (M:134.5)	3.70	2	1.812
Glutathione S-transferase Mu 3	115.2 (M:115.2)	3.41	2	1.833
DNA damage-binding protein 1	154.7 (M:154.7)	4.99	3	1.837
Ig mu chain C region	102.4 (M:102.4)	9.36	2	1.837
Adipocyte plasma membrane-associated protein	203.1 (M:203.1)	4.31	3	1.841
Glycylpeptide N-tetradecanoyltransferase 1	155.3 (M:155.3)	2.93	2	1.849
Lupus La protein	227.5 (M:227.5)	7.58	3	1.869
Transmembrane emp24 domain-containing protein 10	255.7 (M:255.7)	8.64	3	1.886
GMP synthase [glutamine-hydrolyzing]	67.3 (M:67.3)	13.66	2	1.902
Adipocyte enhancer-binding protein 1	685.4 (M:685.4)	7.75	6	1.906
ADP/ATP translocase 2	227.5 (M:227.5)	6.42	4	1.906
Proteasome subunit beta type-3	111.4 (M:111.4)	6.67	2	1.906
Heterogeneous nuclear ribonucleoprotein L	411.4 (M:411.4)	6.82	3	1.922
60S ribosomal protein L7	146.0 (M:146.0)	6.03	3	1.927
NADH-ubiquinone oxidoreductase 75 kDa subunit, mitoc	70.4 (M:70.4)	6.87	2	1.943
Chloride intracellular channel protein 4	285.6 (M:285.6)	3.74	3	1.951
Myeloperoxidase	123.5 (M:123.5)	12.84	2	1.951
PDZ and LIM domain protein 3	158.2 (M:158.2)	4.01	2	1.963
Keratin, type I cytoskeletal 10	261.3 (M:261.3)	4.98	3	1.967
Spermidine synthase	94.3 (M:94.3)	4.30	2	1.996
Ras-related protein Rap-1b	171.7 (M:171.7)	2.92	3	2.049
Tenascin	2462.5 (M:2462.5)	9.41	25	2.049
Dihydrolipoyllysine-residue succinyltransferase compone	94.2 (M:94.2)	4.35	2	2.078
Thrombospondin-1 precursor - Homo sapiens	1501.7 (M:1501.7)	6.00	13	2.102
Dolichyl-diphosphooligosaccharide--protein glycosyltran	196.1 (M:196.1)	10.19	2	2.147
HIV Tat-specific factor 1	261.4 (M:261.4)	2.36	3	2.171
Transferrin receptor protein 1	125.6 (M:125.6)	5.05	2	2.204
40S ribosomal protein S15a	91.2 (M:91.2)	8.27	2	2.29
Keratin, type I cytoskeletal 17	374.0 (M:374.0)	13.17	5	2.31
Serine/threonine-protein phosphatase 2A 65 kDa regulato	195.2 (M:195.2)	9.01	3	2.318

Table S1 DEPs identified by iTRAQ and tandem MS quantification.

Table S1 Proteomic profiling of ESCC by iTRAQ and tandem MS quantification

Protein	Scores	RMS90 [ppm]	Peptides	Ratio (ESCC:T)
SH3 domain-binding glutamic acid-rich-like protein	166.1 (M:166.1)	5.21	2	2.327
DNA replication licensing factor MCM4	275.9 (M:275.9)	5.30	3	2.359
60S acidic ribosomal protein P2	178.1 (M:178.1)	5.41	2	2.392
Versican core protein	253.6 (M:253.6)	12.64	3	2.433
Plakophilin-3	75.0 (M:75.0)	9.09	2	2.465
Alcohol dehydrogenase [NADP+]	150.6 (M:150.6)	5.93	2	2.514
NADH-cytochrome b5 reductase 3	263.1 (M:263.1)	3.71	3	2.518
Proteasome subunit alpha type-5	227.6 (M:227.6)	7.40	3	2.522
Lysosome-associated membrane glycoprotein 2	106.0 (M:106.0)	11.35	2	2.539
Protein S100-A11	336.8 (M:336.8)	7.86	4	2.567
T-complex protein 1 subunit epsilon	158.3 (M:158.3)	14.81	2	2.576
40S ribosomal protein SA	216.0 (M:216.0)	10.44	2	2.641
26S protease regulatory subunit 4	87.9 (M:87.9)	10.37	2	2.645
Serpin H1	162.5 (M:162.5)	4.04	2	2.714
ADP-ribosylation factor-like protein 8B	80.9 (M:80.9)	8.53	2	2.906
Cathepsin B	314.3 (M:314.3)	4.31	2	2.967
EF-hand domain-containing protein D2	143.9 (M:143.9)	9.60	2	3.273
Transgelin-2	289.3 (M:289.3)	7.49	3	3.367
Nicotinamide N-methyltransferase	222.1 (M:222.1)	14.89	2	4.796
**Down-regulated proteins identified by iTRAQ and MS/MS quantification**				
Cornulin	2776.2 (M:2776.2)	5.36	19	0.114
Cystatin-B	236.0 (M:236.0)	10.73	5	0.127
Filaggrin	214.0 (M:214.0)	5.57	3	0.171
Protein-glutamine gamma-glutamyltransferase E	783.0 (M:783.0)	5.08	7	0.2
Serine protease inhibitor Kazal-type 5	872.2 (M:872.2)	7.37	10	0.208
RNA-binding protein Raly	213.9 (M:213.9)	13.68	3	0.233
Selenium-binding protein 1	147.5 (M:147.5)	6.34	2	0.241
Zinc finger protein 185	489.4 (M:489.4)	7.25	4	0.257
Small proline-rich protein 3	350.2 (M:350.2)	9.56	5	0.265
Alpha-2-macroglobulin-like protein 1	617.8 (M:617.8)	5.86	6	0.278
Protein-glutamine gamma-glutamyltransferase K	227.2 (M:227.2)	2.00	3	0.286
Cellular retinoic acid-binding protein 2	343.1 (M:343.1)	7.91	4	0.302
Interleukin-1 receptor antagonist protein	113.9 (M:113.9)	10.09	2	0.306

Table S1 DEPs identified by iTRAQ and tandem MS quantification.

Table S1 Proteomic profiling of ESCC by iTRAQ and tandem MS quantification

Protein	Scores	RMS90 [ppm]	Peptides	Ratio (ESCC:T)
Aquaporin-1	233.6 (M:233.6)	10.50	2	0.31
Cornifin-A	267.8 (M:267.8)	6.90	5	0.31
Mucin-5B	254.4 (M:254.4)	3.26	3	0.327
Myosin light chain kinase, smooth muscle	755.3 (M:755.3)	3.64	7	0.331
Tropomyosin beta chain	1182.6 (M:1182.6)	7.06	18	0.331
Glyoxylate reductase/hydroxypyruvate reductase	239.6 (M:239.6)	6.87	2	0.343
A-kinase anchor protein 12	68.3 (M:68.3)	4.05	2	0.347
Cystatin-A	372.8 (M:372.8)	3.26	5	0.351
65 kDa Yes-associated protein	203.2 (M:203.2)	3.03	2	0.363
Thioredoxin	285.7 (M:285.7)	8.81	5	0.363
Na(+)/H(+) exchange regulatory cofactor NHE-RF1	459.8 (M:459.8)	3.84	4	0.376
Aldehyde dehydrogenase, mitochondrial precursor	182.4 (M:182.4)	6.10	2	0.38
Involucrin	412.7 (M:412.7)	7.04	6	0.38
Tropomyosin alpha-1 chain	1075.5 (M:1075.5)	7.48	17	0.392
Eukaryotic translation initiation factor 2 subunit 1	293.5 (M:293.5)	9.55	3	0.4
Cell surface glycoprotein MUC18	175.0 (M:175.0)	6.72	2	0.404
Annexin A1	1217.4 (M:1217.4)	7.05	12	0.412
Heat shock protein beta-1	909.3 (M:909.3)	5.92	10	0.416
cAMP-dependent protein kinase type II-beta regulatory su	226.0 (M:226.0)	5.59	2	0.429
Cysteine and glycine-rich protein 1	262.4 (M:262.4)	11.77	4	0.437
Fatty acid-binding protein, epidermal	989.2 (M:989.2)	8.25	11	0.453
Ig lambda chain C regions	236.7 (M:236.7)	6.51	3	0.453
Nucleobindin-2	115.9 (M:115.9)	6.85	2	0.457
Serpin B3	380.5 (M:380.5)	3.39	5	0.457
Fibrinogen alpha chain	622.7 (M:622.7)	3.93	6	0.461
Keratin, type I cytoskeletal 13	559.8 (M:559.8)	10.11	7	0.465
Bactericidal/permeability-increasing protein-like 1	100.0 (M:100.0)	0.62	2	0.469
Granulins	145.3 (M:145.3)	10.22	2	0.478
Calpastatin	446.7 (M:446.7)	7.45	5	0.482
Interleukin enhancer-binding factor 2	278.6 (M:278.6)	7.36	2	0.482
Latent-transforming growth factor beta-binding protein 4	215.5 (M:215.5)	1.94	2	0.482
Laminin subunit gamma-1	498.0 (M:498.0)	16.84	5	0.486
Ly6/PLAUR domain-containing protein 3	169.2 (M:169.2)	8.27	3	0.486
Complement C5 precursor	134.2 (M:134.2)	14.04	2	0.494

Table S1 DEPs identified by iTRAQ and tandem MS quantification.

Table S1 Proteomic profiling of ESCC by iTRAQ and tandem MS quantification

Protein	Scores	RMS90 [ppm]	Peptides	Ratio (ESCC:T)
KN motif and ankyrin repeat domain-containing protein 2	153.1 (M:153.1)	13.06	2	0.498
Structural maintenance of chromosomes protein 3	128.2 (M:128.2)	6.52	2	0.498
Serpin B4	82.6 (M:82.6)	8.87	2	0.502
Periplakin	240.9 (M:240.9)	3.25	4	0.506
Protein phosphatase 1 regulatory subunit 12C	116.6 (M:116.6)	7.67	2	0.506
Four and a half LIM domains protein 1	120.1 (M:120.1)	5.18	2	0.51
Tight junction protein ZO-2	168.7 (M:168.7)	12.00	2	0.522
Transcription factor BTF3	268.4 (M:268.4)	14.37	2	0.522
Guanylate-binding protein 6	248.9 (M:248.9)	8.31	5	0.531
Phosphatidylinositide phosphatase SAC1	129.7 (M:129.7)	8.75	2	0.531
von Willebrand factor	79.8 (M:79.8)	0.99	2	0.531
DNA-binding protein A	253.4 (M:253.4)	5.86	2	0.535
Inter-alpha-trypsin inhibitor heavy chain H4	174.4 (M:174.4)	8.47	2	0.535
Keratin, type II cytoskeletal 7	273.0 (M:273.0)	9.86	4	0.535
Phosphoglucomutase-like protein 5	176.6 (M:176.6)	3.43	3	0.535
Tropomyosin alpha-3 chain	764.8 (M:764.8)	8.04	13	0.535
14-3-3 protein zeta/delta	822.3 (M:822.3)	11.40	9	0.539
Thioredoxin-like protein 1	301.8 (M:301.8)	9.07	3	0.539
Ubiquitin-associated protein 2-like	196.1 (M:196.1)	11.90	3	0.539
Transthyretin	323.2 (M:323.2)	3.14	3	0.543
ERO1-like protein alpha	506.8 (M:506.8)	7.67	3	0.547
Protein S100-A9	380.8 (M:380.8)	9.30	4	0.547
Tropomyosin alpha-4 chain	1147.3 (M:1147.3)	8.20	18	0.547
CD5 antigen-like	192.1 (M:192.1)	15.75	2	0.555
Afadin	168.5 (M:168.5)	17.98	2	0.563
Thrombospondin-4	119.2 (M:119.2)	8.56	2	0.563
Annexin A8-like protein 2	278.2 (M:278.2)	13.20	2	0.567
Carboxylesterase 2	74.7 (M:74.7)	6.94	2	0.567
Creatine kinase B-type	709.6 (M:709.6)	10.52	7	0.567
Filamin-A	7289.8 (M:7289.8)	6.45	75	0.567
Ceruloplasmin	707.9 (M:707.9)	8.54	7	0.571
Integrin beta-1	312.0 (M:312.0)	13.05	5	0.58
Actin, aortic smooth muscle	2064.8 (M:2064.8)	6.21	21	0.584
Dynactin subunit 1	279.3 (M:279.3)	5.45	2	0.584

Table S1 DEPs identified by iTRAQ and tandem MS quantification.

Table S1 Proteomic profiling of ESCC by iTRAQ and tandem MS quantification

Protein	Scores	RMS90 [ppm]	Peptides	Ratio (ESCC:T)
Extracellular matrix protein 1	174.7 (M:174.7)	2.18	2	0.584
Keratin, type II cytoskeletal 4	211.2 (M:211.2)	12.57	4	0.588
6-phosphogluconate dehydrogenase, decarboxylating	209.6 (M:209.6)	1.18	2	0.596
Plasminogen	227.6 (M:227.6)	4.77	3	0.596
Antithrombin-III	217.9 (M:217.9)	7.11	3	0.604
Apolipoprotein B-100	126.7 (M:126.7)	19.02	2	0.604
Band 3 anion transport protein	153.5 (M:153.5)	3.84	2	0.604
Protein kinase C and casein kinase substrate in neurons pı	154.8 (M:154.8)	4.07	2	0.608
Synemin	264.2 (M:264.2)	8.02	4	0.608
Ig gamma-3 chain C region	492.8 (M:492.8)	6.28	7	0.612
Protein AMBP	321.9 (M:321.9)	6.46	5	0.612
Tensin-1	160.1 (M:160.1)	1.28	2	0.612
Protein unc-84 homolog B	364.0 (M:364.0)	5.18	4	0.616
Trifunctional purine biosynthetic protein adenosine-3	219.2 (M:219.2)	5.23	2	0.616
Coiled-coil domain-containing protein 97	142.7 (M:142.7)	10.07	2	0.62
Gamma-interferon-inducible protein Ifi-16	81.8 (M:81.8)	2.28	2	0.62
Sister chromatid cohesion protein PDS5 homolog A	151.9 (M:151.9)	2.63	2	0.62
Transmembrane protein 165	206.2 (M:206.2)	11.78	2	0.62
Calcineurin-like phosphoesterase domain-containing prot	358.8 (M:358.8)	3.89	3	0.624
Protein AHNAK2	110.3 (M:110.3)	15.70	2	0.624
40S ribosomal protein S17	266.0 (M:266.0)	3.62	3	0.629
Calmodulin-like protein 3	762.7 (M:762.7)	5.57	9	0.633
Mitochondrial import receptor subunit TOM70	82.0 (M:82.0)	15.62	2	0.637
Neuroblast differentiation-associated protein AHNAK	3418.4 (M:3418.4)	7.99	35	0.637
Transgelin	372.8 (M:372.8)	2.25	6	0.641
Annexin A11	178.8 (M:178.8)	8.66	3	0.645
Ezrin	239.7 (M:239.7)	11.33	5	0.649
Prothrombin	301.9 (M:301.9)	9.85	4	0.649
Ladinin-1	289.3 (M:289.3)	6.16	4	0.653
Prostaglandin reductase 1	73.7 (M:73.7)	12.71	2	0.653
Protein DJ-1	191.6 (M:191.6)	11.71	2	0.653
Fatty aldehyde dehydrogenase	115.0 (M:115.0)	7.38	2	0.657
Peptidyl-prolyl cis-trans isomerase A	365.4 (M:365.4)	6.62	6	0.657
28 kDa heat- and acid-stable phosphoprotein	138.3 (M:138.3)	12.71	2	0.661

Table S1 DEPs identified by iTRAQ and tandem MS quantification.

Table S1 Proteomic profiling of ESCC by iTRAQ and tandem MS quantification

Protein	Scores	RMS90 [ppm]	Peptides	Ratio (ESCC:T)
E3 SUMO-protein ligase RanBP2	137.2 (M:137.2)	9.49	2	0.661
Eukaryotic translation initiation factor 3 subunit C	223.4 (M:223.4)	2.88	2	0.661
EH domain-containing protein 2	271.0 (M:271.0)	14.86	3	0.665
Fibrinogen gamma chain precursor - Homo sapiens (Hum	187.5 (M:187.5)	7.90	3	0.665
Ig alpha-1 chain C region	417.0 (M:417.0)	6.95	5	0.665
Ig gamma-1 chain C region	831.4 (M:831.4)	7.75	8	0.665
Scaffold attachment factor B1	370.2 (M:370.2)	6.16	5	0.665
Caldesmon	935.7 (M:935.7)	5.84	11	0.669
Heat shock 70 kDa protein 1	1408.6 (M:1408.6)	8.18	15	0.669
Malate dehydrogenase, cytoplasmic	277.8 (M:277.8)	4.84	3	0.669

Table S2 Statistical characteristics of the PPI networks.

Variables	Value	Variables	Value
Nodes	6392	Max degree	568
Edges	22604	Min degree	1
Average degree	7.0726	Average path length	3.6672
Power-law exponent	-1.7770	SW	221.1198
Average clust. Coeff.	0.2003		

Table S2 Statistical characteristics of the PPI networks.

Table S3 Differentially expressed genes identified from GSE 23400.

P value	DEGs	P value	DEGs	P value	DEGs	P value	DEGs	P value	DEGs
0.29	RFC4	0.46	TOPBP1	0.28	SPARC	0.57	CCT3	0.6	RBM28
0.44	CBX3	0.35	MET	0.57	ZNF146	3.82	NUCB2	0.2	MMP12
0.22	ECT2	0.43	HPRT1	0.38	MCM4	0.54	SUPT16H	0.5	DERL1
0.12	COL1A1	0.55	LSM4	0.36	CCNB1	0.57	ADAR	0.56	CKAP2
0.044	MMP1	0.3	ASPM	0.42	TRIP13	0.35	ITGA6	1.92	LRRFIP2
0.3	MFAP2	0.56	HSP90AA1	0.51	SNRPG	0.49	SLC38A6	1.7	ST3GAL4
0.37	KIF4A	2.38	GPD1L	0.33	HMGB3	3.01	MGLL	0.58	NONO
0.33	CKS1B	0.5	DKC1	18.32	CRISP3	0.6	TM9SF1	1.77	NCOA1
0.11	SPP1	0.53	PSME4	0.46	ATP6V1C1	15.1	MAL	2.25	ECHDC2
0.4	MCM6	0.46	BUB1	0.46	GMPS	0.56	WHSC1	0.58	M6PR
0.36	MCM2	0.38	LAPTM4B	0.46	AURKB	0.47	CHSY1	0.28	TNC
0.24	PLAU	0.5	HOXB7	0.56	HSP90B1	0.41	SLC16A1	0.55	MEX3D
0.47	AGRN	0.55	CPSF6	0.52	NCAPG2	0.57	PSMB4	7.46	SLURP1
0.34	BUB1B	0.36	NETO2	0.23	COL5A2	0.52	PRPF4	0.29	CDH3
0.39	KIF14	0.39	CDKN3	0.34	SERPINH1	0.53	RPL39L	0.43	GGH
0.33	GINS1	0.47	PTDSS1	4.46	HPGD	0.32	MMP11	0.48	PUS7
0.5	BID	2.44	SORBS2	0.42	PRKDC	0.42	ZWINT	0.47	PARP12
0.33	CDK1	0.57	ADSS	6.49	PPP1R3C	0.47	E2F3	0.55	DEK
0.51	NUP155	0.24	TOP2A	0.55	H2AFV	0.54	HSP90AB1	0.5	MCM10
0.54	ATP2C1	0.52	SNRPE	0.51	MRPL3	0.4	SLC25A32	0.4	DSG2
0.27	CEP55	0.38	MARCKSL1	0.46	SLC33A1	0.42	HAT1	0.41	FAP
0.55	PDIA6	0.21	COL3A1	0.53	NCAPD2	2.52	SASH1	0.56	TMX1
0.21	SNAI2	0.46	FEN1	0.56	AHSA1	0.3	COL1A2	4.18	GPX3
0.53	ACLY	0.21	VCAN	4.33	ADH1B	0.49	HOMER3	2.5	ABLIM3
0.44	ITPR3	2.42	UBL3	0.37	BIRC5	0.54	PSMA3	0.61	MYH9
0.39	PLXNA1	0.4	AURKA	0.33	NUSAP1	2.45	GDPD3	1.94	COBL
0.41	ACTL6A	4.12	CFD	0.5	YEATS2	6.6	ENDOU	0.57	KIAA0907
0.31	FSCN1	0.53	ANP32E	0.42	HSPH1	0.59	GTF3C3	0.52	RAD21
0.53	RPN1	0.33	CDC20	0.53	TIMELESS	0.51	XPOT	0.48	FXR1
0.39	UBE2C	0.45	SLC20A1	0.31	PRC1	0.56	ILF3	0.37	PBK
0.49	KIF2C	0.39	NEK2	0.56	FTSJ2	0.39	RAI14	0.47	RACGAP1
0.36	DLGAP5	0.4	TPX2	0.41	ATAD2	0.55	LMNB2	0.55	NCBP2
0.28	SOX4	0.39	GMNN	0.5	RRM1	0.38	BGN	0.47	LRP12
0.4	CENPF	0.52	ARL6IP1	0.47	TMEM185B	0.27	TGFBI	0.53	NASP
0.48	PTK7	0.35	TTK	0.52	PTK2	0.54	P4HA1	2.12	SELENBP1
0.46	RANBP1	0.53	GPR137B	0.46	DPY19L4	0.59	TAF2	0.57	MGAT2
0.45	DNMT1	0.4	PCNA	0.4	MELK	0.55	METTL3	10.36	CLCA4
0.53	NUDT1	0.52	HSPD1	0.51	POLR2K	0.53	LSM5	0.38	FST
0.36	COL7A1	0.5	HEATR1	0.34	IGF2BP2	0.46	LMNB1	0.35	COL6A3
0.34	DTL	0.57	SNRPB	0.5	SAC3D1	0.51	TGIF1	0.52	NOP56
0.19	CDH11	0.49	OLFML2B	0.41	MAD2L1	2.08	SH3GLB2	0.59	ATP13A3
0.46	FANCI	0.55	POLR2H	0.41	MCM5	0.45	ARPC1B	0.29	LAMC2
0.44	KIF20A	0.56	CCT5	0.46	LRRC8D	2.41	RMND5B	2.21	GALNT12
0.5	RUVBL1	0.28	LAMB3	0.18	COL11A1	0.59	KDELR3	0.58	SRPK2
0.47	ATR	0.49	CDK4	0.38	SMC2	0.52	NME1	2.16	FAM107A
0.33	MEST	0.45	TBL1XR1	0.55	GSTO1	0.62	SRSF10	0.58	RRP15
0.32	FZD6	0.34	THBS2	0.5	ILF2	0.58	FANCL	0.37	FNDC3B
0.44	CENPA	0.29	SULF1	0.5	RFC3	0.42	ITGB4	0.54	PFDN2
0.45	EFNA1	3.36	KAT2B	0.45	NUP107	0.54	TUBB	0.55	PDIA5
1.79	CRYL1	2.3	KIAA0232	0.57	RPA3	0.49	MCM3	0.51	FAM60A
0.12	POSTN	2.76	SIM2	0.41	VOPP1	0.47	JAG2	0.53	GINS2
0.33	FOXM1	0.46	HSPE1	0.36	MTHFD2	2.17	PADI1	0.51	BAZ1A
0.25	CKS2	0.46	RAD51AP1	3.48	LPIN1	0.42	TYMS	0.56	UCK2
0.38	TFRC	0.46	MSH6	0.46	MYO10	0.53	KIF18B	0.52	PPT1

Table S3 Differentially expressed genes identified from GSE 23400.

P value	DEGs	P value	DEGs	P value	DEGs	P value	DEGs	P value	DEGs
9.7	TGM3	0.56	UMPS	0.45	SLC2A1	0.51	TMEM97	4.39	ALOX12
0.46	ATP1B3	0.56	CEP170	0.35	APOE	0.52	CTSK	0.52	AGPS
0.47	KIF11	0.56	DNAJA1	0.55	RAN	1.73	MGST2	1.87	ZFP36
0.6	RNF7	2	ALDH9A1	0.57	TMF1	0.54	IVNS1ABP	0.6	RBBP8
0.58	RPA1	0.57	FTL	0.6	MTDH	0.59	ARPP19	0.48	AMIGO2
0.56	ELF4	0.48	PAICS	4.92	ECM1	0.6	CCNA2	1.72	SHMT1
3.42	CLEC3B	1.69	HIGD1A	0.47	MCM7	0.63	USP1	0.61	UPF3B
0.42	SLC39A14	6.47	SPINK5	2	PMM1	0.43	TIMP1	0.56	PTTG1
2.02	ZNF426	0.41	COL10A1	3.52	EPS8L1	4.54	HOPX	1.59	SUCLG2
0.59	DNAJC9	0.57	MAGOH	1.81	WFDC1	2.7	C2orf54	1.9	LRP10
2.13	NDRG2	0.55	STK3	0.53	PLOD1	2.12	SORT1	2.35	RANBP9
0.52	EZH2	1.64	LMBRD1	0.36	HEY1	0.52	LPCAT1	0.61	IKBKAP
1.99	AGFG2	0.52	RCN1	0.58	ARMC1	2.62	CYP2C18	0.44	MLF1
0.54	GTPBP4	3.74	FMO2	1.9	TOB1	2.09	PHACTR4	0.58	RRAS2
0.48	SNAPC1	1.99	CPEB3	0.6	SELT	3.21	IL18	0.66	SRPRB
0.61	PSMD14	0.41	KIAA0101	0.51	ELOVL5	0.55	LY6E	2.88	GLTP
2.57	FUT6	0.5	KIAA1598	0.4	TUSC3	2.37	PLAGL1	0.53	CEBPB
0.55	SLC3A2	2.13	ABHD5	0.56	PFDN4	2.34	ITM2A	4.11	TGM1
0.51	CDC6	0.58	BANF1	0.49	MSN	5.21	FLG	0.64	NOC3L
0.32	COL5A1	0.44	CDC25B	2.96	UPK1A	5.88	KRT4	0.31	ODC1
0.51	MEM194	0.47	IFI16	0.33	LAMA3	0.53	MYO1B	0.26	ISG15
0.56	SLC5A3	0.43	MFHAS1	2.71	ZNF365	2.48	PDLIM2	0.55	TNFRSF10E
2.08	ECISBP2	0.53	IFI30	0.58	NUP85	0.61	TMPO	0.55	MRPS12
0.39	LOXL2	0.47	PTPRK	0.52	PDPN	1.98	ANXA11	1.69	SH3GL1
0.58	HOXC10	2.93	CEACAM1	0.47	SH3BP4	3.49	CRABP2	2.17	RORA
0.56	CENPN	0.58	C5orf15	0.57	LRP8	0.5	ITGAV	0.61	MBD4
0.56	EEF1E1	0.52	SHMT2	0.5	MYH10	0.63	CALU	2.5	CWH43
0.56	THY1	0.46	MXRA5	0.48	LOX	3.45	MXD1	5.97	RHCG
0.58	CCT6A	0.62	RBBP4	0.59	MTHFD1	0.26	INHBA	1.83	TJP1
0.57	RFC5	0.55	ACVR1	0.28	EPCAM	0.57	MTFR1	0.59	ACOT13
0.58	H2AFZ	2.96	ID4	0.28	CST1	3.98	PPL	2.03	ACOX1
3.02	HSPB8	0.56	NUP37	0.6	NMD3	0.37	LUM	1.81	TJP3
3.1	SCNN1B	0.45	VRK1	2.57	TNXB	0.58	TRAM2	0.21	PTHLH
12.15	CRNN	0.51	BTG3	1.68	ITIH5	0.57	MRPL13	1.79	TAX1BP1
0.55	CSE1L	4.62	PSCA	0.59	CNIH4	2.73	CRYAB	3.85	BLNK
0.6	ADO	0.62	DDX18	2	ACADM	1.81	ACAA1	0.69	ZBTB1
3.23	ABLIM1	1.96	ZBTB16	8.31	TMPRSS11	3.45	C1orf116	2.62	GMDS
6.77	KLK13	0.52	PLAUR	4.81	EMP1	2.6	TP53I3	0.55	CCT2
0.56	ANKLE2	4.07	TMPRSS2	0.54	ABCA1	1.59	VAT1	0.65	RFWD3
0.4	SLC39A6	10.89	SCEL	0.51	EXT1	1.66	SLC16A7	1.79	TTC39A
0.38	SERPINE1	1.72	SYNGR1	0.56	ASF1A	0.6	PRPS2	1.93	RRAGD
2.33	TRIP10	2.44	KANK1	0.36	TP63	1.67	PTGDS	0.61	IRF9
2.18	AHNAK	0.55	ZWILCH	0.6	MRPS28	0.47	MMD	0.45	COL4A1
0.49	KPNA2	7.27	CLIC3	2.27	HLF	2.23	ANXA9	3.5	DUSP5
0.5	CCNB2	0.6	UNG	3.78	PRSS3	1.56	PPP1CB	1.79	WWC1
0.52	ENY2	0.59	RCN2	0.35	MMP9	0.55	SKP2	0.66	KTN1
2.92	OBFC1	3.13	TTC9	0.58	CBX1	0.52	TAF1D	0.58	GMFB
2.28	CXCR2	0.59	TSR1	0.34	TMEM45A	2.61	EPS8L2	2.28	FUT3
0.48	DFNA5	0.55	FERMT1	1.66	RAB5A	1.84	PDCD4	0.52	P4HA2
0.52	SNRPD1	3.19	EHD3	0.5	TRIB2	2.3	CITED2	3.16	KLK12
0.47	BOP1	0.41	ENAH	8.71	CRCT1	2.05	C18orf25	0.42	CTSC
0.41	HLTF	0.54	EIF2AK2	0.19	KRT17	0.6	H2AFX	0.49	PANX1
1.99	CES2	0.58	MMP14	0.52	PAK2	0.54	ADORA2B	1.84	AMOTL2
2.34	STK39	0.49	HMMR	2.18	KLF4	2.96	SULT2B1	0.55	HOXC6
0.49	UBE2S	0.39	GPNMB	0.42	PXDN	0.45	IRS1	1.67	MLLT4
0.29	IGFBP3	2.22	PHACTR2	0.51	CBLB	0.43	PMEPA1	2.04	VPS37B

Table S3 Differentially expressed genes identified from GSE 23400.

P value	DEGs	P value	DEGs	P value	DEGs	P value	DEGs	P value	DEGs
0.64	CBFB	1.75	CDKN2AIP	2.17	SLK	2.45	YOD1	0.64	C1QBP
3.13	CYP4B1	0.52	AKR1B1	4.77	KRT13	2.35	LDB3	3.69	SPRR2C
0.42	RRM2	0.67	SMC3	1.72	KLHL2	2.2	CYP3A5	1.69	PALMD
2.38	GYS2	0.61	TUBG1	0.21	MMP10	0.4	MMP13	0.64	ACOT9
1.89	NAGK	2.21	TGFBR3	0.65	CD164	1.71	TICAM1	0.59	STK17A
2.46	PCP4	0.44	PRSS23	0.21	MAGEA3	2.02	ESPL1	2.21	SMAGP
2.05	DHRS1	2.84	CXCL12	1.99	RRAD	4.01	SERPINB13	0.42	IGF2BP3
2.29	METTL7A	2.14	CAMK2N1	0.63	STC1	1.9	USP6NL	3.87	SERPINB2
1.57	TOLLIP	2.02	NPEPPS	2.3	ACPP	0.61	SAV1	1.77	TECR
2.5	PDZD2	0.43	PPFIA1	2.4	RCAN2	1.61	DTX2	1.8	GALE
2.74	PAX9	0.66	RHEB	1.73	FBXO3	1.54	C12orf29	1.74	RASAL2
1.79	RASAL1	0.47	NNMT	3.34	PITX1	0.66	IPO7	1.63	CUL3
0.6	YWHAH	0.51	IFITM1	0.64	PHB	0.53	LAMB1	0.57	TNFAIP3
0.62	COL18A1	0.39	PFN2	0.51	IFI35	0.6	IFITM3	0.62	COX17
1.7	PPDPF	0.61	UTP18	0.51	STAT1	1.62	CCDC6	0.54	APOL1
0.56	SLC1A4	0.54	STEAP1	0.66	OGT	0.53	ABCC5	0.42	PTPRZ1
2.5	BEX4	3.44	NMU	2.25	RIOK3	0.67	MLEC	1.72	EPHA1
1.81	SERPINB6	0.47	TNFAIP6	0.21	MAGEA6	0.65	HDGFRP3	0.47	IFI44
0.41	FAT1	1.49	CYP2C9	0.58	PSME2	1.69	GCHFR	0.42	FADS1
1.69	SPAG16	3.94	SERPINB1	0.52	MMP2	0.66	CORO1C	0.63	CTSB
0.58	MTA1	0.53	PPAP2C	1.73	TMEM40	0.6	DNM1	1.78	INPP1
1.65	ACOX3	2.69	UPK3B	1.84	ETHE1	2.12	DOCK9	3.58	SERPINB3
0.66	ZNF131	2.35	DPT	0.62	STRN3	0.49	WARS	0.68	CLNS1A
0.44	IFI6	2.2	PTN	2.09	PDZRN3	2.01	CNN3	0.55	UBE2L6
0.6	6GALNA	2.28	MAFF	1.82	GULP1	0.58	LDHB	2.38	RBM47
3.64	ZNF185	1.74	LTA4H	0.66	GLIPR1	0.67	DMXL2	0.47	PSMB9
2.88	CCNG2	2.46	TMOD3	1.96	CRIP2	1.56	BSPRY	2.11	C7
0.59	G3BP1	2.15	SLC24A3	1.51	HSD17B4	2.08	OR7E14P	1.74	UBE2G1
3.81	GABRP	1.92	HEBP2	1.58	ABCD3	2.85	KLK11	2.18	ELOVL6
0.61	HEXB	0.43	FADD	1.84	RAB11A	1.91	TST	2.1	NEBL
0.64	ABCG1	1.7	ITSN2	0.57	CD44	0.62	SLC7A8	0.66	YBX1
0.62	NIT2	0.48	SSFA2	1.97	DYNLT3	1.54	PPP1R7	1.9	ARHGAP32
0.6	RIN2	2.02	CYP2E1	0.57	SLC6A8	0.65	EGFL6	1.34	ECHDC3
2.55	EVPL	0.64	PIK3CA	0.58	TCFL5	1.85	TF	1.62	GADD45B
0.64	ZNF532	0.61	PON2	1.52	ATP10B	2.59	DUOX1	1.55	TSPO
0.34	ASPN	0.64	RPS21	0.62	GNG10	0.41	MMP3	2.05	ATP1A2
0.4	BASP1	1.84	RNASE4	4.28	CEACAM7	0.6	UBXN7	3.13	SPRR3
3.26	MALL	0.64	SLC38A2	2.31	ERO1L	0.41	ALCAM	1.55	CLCN3
1.99	PIM1	2.52	ABCA8	1.6	CALM1	2.1	FRMD4B	0.55	DDX60
0.59	TDP2	1.66	PLEKHM1	0.43	NELL2	1.93	HS3ST1	2.67	ZNF750
3.62	MPRSS11	3.8	IL1RN	1.7	MECOM	0.66	SMC1A	0.61	LPGAT1
0.6	SLC39A4	0.55	HTRA1	1.88	RNF141	1.58	CH25H	0.65	RNF13
1.83	MEIS1	1.92	BLVRB	1.83	LMO2	1.53	ALDH2	1.59	ARF6
0.64	TRIO	0.51	HMGB2	1.99	FUT2	0.71	MAP4K5	1.98	PELI1
2.05	CAST	1.74	DUSP1	0.58	CCNB1IP1	0.5	LY96	1.54	PRDM1
0.59	POLE3	3.91	CEACAM5	0.57	GLS	0.67	HMGB1	0.73	PTBP1
1.81	COBLL1	0.62	NOL7	0.66	TCERG1	1.58	TOM1	1.65	MAOB
0.63	GALNAC	0.64	GNS	1.61	ATP6V1D	0.61	COTL1	0.68	CHN1
2.85	AIM1L	0.65	SDF4	2.05	ANXA3	0.61	EIF4EBP1	1.42	RAP1A
2.03	NBEAL2	0.42	SFRP4	0.5	DST	1.72	VPS4B	1.57	ALDH3A2
0.6	BTG1	2.09	EXPH5	2.21	ZDHHC13	0.57	KCNS3	0.65	SP100
0.45	PLOD2	1.89	ZFAND5	0.65	SPON2	3.57	CEACAM6	0.65	TGFB1
1.89	PER2	1.75	DMXL1	3.14	EHF	2.64	PTK6	0.48	FN1
0.55	IFNGR1	1.82	TM7SF2	0.55	CLDN1	0.58	EPB41L4B	1.71	TJP2
2.12	EPB41L3	0.58	TMEM123	1.97	ERBB3	0.68	NUP50	1.46	CFLAR
0.65	SMURF2	0.69	ANXA5	1.85	MAOA	4.15	MUC5B	1.54	SH3GLB1

Table S3 Differentially expressed genes identified from GSE 23400.

P value	DEGs	P value	DEGs	P value	DEGs	P value	DEGs	P value	DEGs
1.5	NPAS2	2.1	TIAM1	0.63	PYCARD	1.48	AGA	0.45	NTRK2
0.55	FARP1	0.64	ΓNFRSF12A	1.53	WDR26	2.22	MYH11	2.11	KLK7
3.15	PLAC8	1.59	XK	1.58	GSN	1.54	PPFIBP2	0.59	TMEM158
1.58	MAST4	2.82	SCNN1A	2.78	EREG	1.41	RUFY3	1.68	ZSCAN18
1.92	CLDN10	0.49	FOXE1	0.69	HLA-B	0.59	RSAD2	0.46	BST2
2.03	CHRDL1	1.5	CHMP2B	0.64	GNAI1	0.76	PTMA	0.51	CXCL10
1.84	COL14A1	0.58	PRNP	0.7	LGALS8	0.69	MACF1	0.63	HIST1H2BC
0.59	NDRG1	0.37	ANO1	1.47	PTPN3	1.71	DAAM2	1.35	JMJD7
0.59	NMI	1.51	TSC22D3	1.93	CD24	0.69	NGRN	1.48	ALDH7A1
0.41	LAMP3	0.46	IFI44L	1.53	NCOA2	0.51	GBP1	1.44	ITPR2
0.63	METTL5	1.53	PBX1	2.1	LYPD3	2.39	PRR4	0.64	SLC16A3
1.64	FBXO34	0.67	AUTS2	1.85	EPHA2	0.63	OAS3	2.01	IVL
0.63	SSR1	0.44	APOBEC3B	0.61	TNS3	1.55	DNASE1L3	1.84	TSPAN6
0.66	ITGB1	0.42	GCLM	1.62	HMOX1	1.97	COX7A1	0.65	UCP2
0.53	C1QB	1.42	GSTT1	1.89	HPSE	0.68	FAM69A	1.67	TPSB2
0.56	NFIL3	2.37	LMOD1	1.47	PDCD6IP	1.47	KDM5D	0.66	SMC4
1.84	ACACB	1.57	CBX7	0.65	HLA-F	0.71	GALNT2	0.54	COL4A2
2.21	FHL1	1.88	DHRS9	1.78	TPPP3	1.95	ATP1B1	0.71	ENO1
1.75	PRSS22	2.26	ELF3	2	ALDH3B2	1.47	ERBB2	0.72	GOLGA8A
0.5	MLLT11	0.66	FKBP11	1.4	ZCCHC6	0.51	LGALS1	1.48	CDS1
1.59	ASCC2	1.27	AMFR	1.52	EZR	1.73	RND3	1.62	RIPK4
1.67	SNX24	0.56	TXNRD1	1.64	SLC26A2	0.61	ISLR	0.47	CXCL14
0.52	TYMP	1.46	PDLIM5	0.54	ENO2	0.66	HCP5	1.5	PLD1
0.6	LYN	0.62	CD163	1.97	CLDN7	1.69	SFRP1	0.73	STAG2
2.2	MUC1	1.75	MAP3K9	0.68	BCL6	0.65	GBAS	1.58	SQRDL
1.69	MFAP4	1.67	STK24	0.65	JAG1	0.75	PPP3CA	2.83	LCN2
1.66	RMND5A	0.63	FAM3C	0.4	NEFL	0.69	ITGA5	0.74	GALNT10
0.7	PRPF4B	1.57	CLTB	0.62	PSMB8	0.46	UCHL1	0.57	TNFSF10
0.58	TYROBP	0.64	DEGS1	0.54	COL17A1	1.6	TIPARP	0.71	PAIP1
1.83	CLDN4	0.53	PSPH	1.51	ITPKC	2.05	MT1M	1.66	PER3
0.48	RBP1	1.53	CD59	0.73	NFATC2IP	2.06	TFF3	1.85	ZBED2
0.57	C1S	0.66	LAP3	1.41	SLC39A2	1.79	NFASC	0.64	PDGFA
2.44	EPHX3	0.67	VEGFA	1.9	AOC3	0.7	IGF1R	1.66	CD55
1.94	RBPMS	0.61	DSE	0.73	REL	1.64	TPSAB1	1.39	ARL6IP5
0.7	RECQL	0.73	GOLGA8B	0.66	GALC	0.65	SQLE	1.67	CSRP1
1.89	MANSC1	1.56	GCNT3	2.28	AIM1	1.9	PRKAR2B	1.46	RAB9A
0.65	CCDC91	0.68	IFITM2	0.57	GCLC	1.44	EPB41L1	1.97	SMTN
1.57	MARCH5	1.68	GATM	1.81	FAM129A	2.5	CNN1	1.82	KLK8
1.96	CSTB	1.62	DDAH1	2.03	AQP1	0.6	LBH	0.68	CCL18
0.53	GABRE	1.54	CLIP1	1.58	SLCO4A1	2.67	SORBS1	0.74	NUDT21
1.92	VAV3	2.05	MYO6	1.44	CPNE3	0.7	CALR	0.71	BTN3A2
0.63	ERLIN2	1.5	CST3	0.6	B4GALT4	0.69	RAC2	2.48	KRT24
1.68	NT5C2	2.17	FOSB	3.16	S100P	0.72	ΓMEM106E	1.6	TPD52L1
0.66	HTATIP2	0.7	HMGN4	0.58	RAB31	0.58	VCAM1	0.58	DUSP6
2.26	CDA	0.56	TAP1	0.7	GPC1	0.43	GREM1	1.71	APOD
0.63	ATP2B1	1.52	PLIN3	0.56	LOXL1	1.63	RASL12	1.96	SAMD9
2.56	₹AB11FIP	0.75	MTPAP	0.63	PLTP	1.99	AZGP1	0.54	SEMA3C
0.65	ΓMEM14A	3.47	SPRR2B	1.59	DIO2	0.69	CD14	1.52	DGKA
0.7	FNTA	1.51	USP46	1.58	UGCG	0.52	IFIT1	1.25	LRRFIP1
2.65	PHLDA1	0.54	DHCR7	3.59	SERPINB4	1.61	PLS1	0.71	FHL2
1.81	EYA2	2.3	S100A14	0.64	SEL1L3	0.62	OAS2	0.71	HLA-C
0.69	SLC25A36	1.53	CELF2	1.69	IL13RA1	1.67	FNBP1	2.17	RAB25
0.63	APP	1.55	CMAS	0.68	CAPRIN2	0.6	SCO2	1.68	PPP1R12B
1.51	₹ABGGTA	0.55	SLC7A5	0.67	OPN3	1.54	KLF2	1.47	GRN
0.65	NID2	1.88	CCDC69	1.55	INPP5A	0.63	CHST15	2.22	MYL9
0.43	CXCL1	1.58	KIT	1.55	KLF6	1.49	MFSD5	1.46	CDKN1C

Table S3 Differentially expressed genes identified from GSE 23400.

P value	DEGs	P value	DEGs	P value	DEGs	P value	DEGs	P value	DEGs
1.39	WNK1	0.68	SCRN1						
0.69	NRP1	0.7	FCHSD2						
0.65	C1QA	1.78	TCN1						
1.42	PLEKHA5	0.71	ELMO3						
1.75	REEP1	2.55	AQP3						
1.4	CPE	1.37	HOMER2						
1.6	ITCH	1.68	CLU						
2	ANXA1	2.08	SYNM						
1.48	OSTF1	1.65	RHOD						
0.73	SEH1L	0.79	ESF1						
1.59	ALDH1A3	1.36	CAPN1						
1.68	PLBD1	1.41	TACC1						
0.7	SLCO3A1	0.72	IGFBP4						
1.42	TMED3	0.73	KRIT1						
0.8	SEC63	1.37	PPP2R2A						
1.38	RREB1	0.77	RANBP2						
0.61	HK2	1.92	IGJ						
0.69	IPO5	1.39	PPAP2B						
0.63	RARRES1	2.27	KRT15						
1.56	CADM1	0.66	COL16A1						
0.62	AEBP1	0.59	PLAT						
1.69	FLNC	0.55	GPX2						
1.4	DAAM1	0.75	NNT						
0.6	IGFBP7	0.7	CEBPD						
1.73	RAPGEFL	1.66	KCNMB1						
0.7	OSTM1	1.38	ESRP2						
1.4	CARHSP1	0.72	HSD17B11						
1.46	IL6ST	1.42	PADI3						
0.58	CHI3L1	1.38	HMOX2						
0.7	MBNL1	0.68	THBS1						
1.65	RGS5	0.62	G6PD						
1.34	S100A4	1.32	POF1B						
2.26	ACTG2	0.61	F3						
1.3	P2RY2	1.52	KRT31						
1.34	RAP2A	1.3	EIF1AY						
0.65	SEC23A	0.39	KRT14						
1.31	MYCBP2	2.07	PDZK1IP1						
1.84	MPZL2	0.77	SYNCRIP						
1.8	CSTA	0.72	ID3						
2.35	SLPI	1.36	SEMA4D						
0.63	KRT8	1.65	SPINT1						
1.95	ADH7	2.14	S100A9						
0.59	EGFR	1.44	LIMCH1						
0.7	PLSCR1	0.76	COX11						
1.35	RGS12	1.43	SPATA5L1						
1.71	PRSS8	1.65	KCNMA1						
0.72	BMP7	0.74	IRX4						
0.66	KLF10	0.76	ZC3H12A						
1.39	APOLD1	1.45	ZCCHC24						
1.5	ITPR1	0.63	LTBP1						
0.74	DLG1	1.41	LY6G6C						
0.66	GRB10	1.56	DDX3Y						
1.58	SH3YL1								
1.35	ELL2								
0.63	NPL								
1.28	FBXL5								

Table S3 Differentially expressed genes identified from GSE 23400.

Table S4 Overlaping between DEG and DEP.

Ratio of DEGs	DEGs	DEPs	Ratio of DEPs
0.62	AEBP1	AEBP1	1.91
2.18	AHNAK	AHNAK	0.64
1.57	ALDH3A2	ALDH3A2	0.66
2	ANXA1	ANXA1	0.41
1.98	ANXA11	ANXA11	0.64
0.69	ANXA5	ANXA5	1.69
2.03	AQP1	AQP1	0.31
2.05	CAST	CAST	0.48
0.55	CCT2	CCT2	1.68
0.56	CCT5	CCT5	2.58
1.99	CES2	CES2	0.57
1.68	CLU	CLU	1.54
0.12	COL1A1	COL1A1	1.67
0.3	COL1A2	COL1A2	1.56
0.35	COL6A3	COL6A3	1.57
3.49	CRABP2	CRABP2	0.30
12.15	CRNN	CRNN	0.11
1.67	CSRP1	CSRP1	0.44
1.8	CSTA	CSTA	0.35
1.96	CSTB	CSTB	0.13
0.63	CTSB	CTSB	2.97
4.92	ECM1	ECM1	0.58
2.31	ERO1L	ERO1L	0.55
1.52	EZR	EZR	0.65
2.21	FHL1	FHL1	0.51
0.48	FN1	FN1	1.62
0.46	GMPS	GMPS	1.90
1.47	GRN	GRN	0.48
0.47	IFI16	IFI16	0.62
3.8	IL1RN	IL1RN	0.31
0.5	ILF2	ILF2	0.48
0.66	ITGB1	ITGB1	0.58
2.01	IVL	IVL	0.38
4.77	KRT13	KRT13	0.47
0.19	KRT17	KRT17	2.31
5.88	KRT4	KRT4	0.59
0.37	LUM	LUM	1.60
2.1	LYPD3	LYPD3	0.49
0.38	MCM4	MCM4	2.36
1.67	MLLT4	MLLT4	0.56
4.15	MUC5B	MUC5B	0.33
0.46	MXRA5	MXRA5	1.73
0.47	NNMT	NNMT	4.80
0.58	NONO	NONO	1.75
3.82	NUCB2	NUCB2	0.46
0.12	POSTN	POSTN	1.75
3.98	PPL	PPL	0.51
1.9	PRKAR2B	PRKAR2B	0.43
0.77	RANBP2	RANBP2	0.66
1.42	RAP1A	RAP1A	1.67
2.14	S100A9	S100A9	0.55
2.12	SELENBP1	SELENBP1	0.24

Ratio of DEGs	DEGs	DEPs	Ratio of DEPs
3.58	SERPINB3	SERPINB3	0.46
3.59	SERPINB4	SERPINB4	0.50
0.34	SERPINH1	SERPINH1	2.71
6.47	SPINK5	SPINK5	0.21
3.13	SPRR3	SPRR3	0.27
1.58	SQRDL	SQRDL	1.52
0.51	STAT1	STAT1	1.69
2.08	SYNM	SYNM	0.61
0.38	TFRC	TFRC	2.20
9.7	TGM3	TGM3	0.20
1.71	TJP2	TJP2	0.52
0.28	TNC	TNC	2.05
0.21	VCAN	VCAN	2.43
1.72	VPS4B	VPS4B	1.56
3.64	ZNF185	ZNF185	0.26

Table S4 Overlaping between DEG and DEP.

Table S5 Condordance between DEGs and DEPs.

		DEPs		
		Up-regulated	Down-regulated	
DEGs	Up-regulated	22	4	$P = 1\times10^{-12}$
	Down-regulated	4	37	

Table S6 Enriched biological processes and KEGG pathways of SDNs, DEPs, DEGs, and SDN-TNMs.

Enrichment analysis of GO biological processes and KEGG pathways for the signature molecules of SDN-based TNM classifier

GOID	Ontology Source	GOTerm	% Associ	Term P Value	Associated Genes Found
GO:0000184	GO_BiologicalProcess	nuclear-transcribed mRNA catabolic process, nonsense-mediated decay	5.26316	1.30E-11	PPP2R1A, RPL12, RPL3, RPL7, RPLP2, RPS18, RPS6
KEGG:03010	KEGG	Ribosome	4.44444	1.41E-09	RPL12, RPL3, RPL7, RPLP2, RPS18, RPS6
GO:0006413	GO_BiologicalProcess	translational initiation	4.09357	7.72E-11	KHDRBS1, RPL12, RPL3, RPL7, RPLP2, RPS18, RPS6
GO:0006414	GO_BiologicalProcess	translational elongation	4.95868	7.25E-10	RPL12, RPL3, RPL7, RPLP2, RPS18, RPS6
GO:0006415	GO_BiologicalProcess	translational termination	5.94059	2.41E-10	RPL12, RPL3, RPL7, RPLP2, RPS18, RPS6
GO:0006613	GO_BiologicalProcess	cotranslational protein targeting to	5	6.89E-10	RPL12, RPL3, RPL7, RPLP2, RPS18, RPS6
GO:0006614	GO_BiologicalProcess	SRP-dependent cotranslational protein targeting to membrane	5.08475	6.22E-10	RPL12, RPL3, RPL7, RPLP2, RPS18, RPS6
GO:0019083	GO_BiologicalProcess	viral transcription	4.05405	2.45E-09	RPL12, RPL3, RPL7, RPLP2, RPS18, RPS6
GO:0045047	GO_BiologicalProcess	protein targeting to ER	5	6.89E-10	RPL12, RPL3, RPL7, RPLP2, RPS18, RPS6
GO:0070972	GO_BiologicalProcess	protein localization to endoplasmic reticulum	4.41176	1.47E-09	RPL12, RPL3, RPL7, RPLP2, RPS18, RPS6
GO:0072599	GO_BiologicalProcess	establishment of protein localization to endoplasmic reticulum	4.95868	7.25E-10	RPL12, RPL3, RPL7, RPLP2, RPS18, RPS6
**Enrichment analysis of GO biological processes and KEGG pathways for top 50 DEPs.**					
GO:0009913	GO_BiologicalProcess	epidermal cell differentiation	6.13497	3.02E-11	CSTA, FLG, IVL, KRT17, SPINK5, SPRR1A, SPRR3, TGM1, TGM3, YAP1
GO:0018149	GO_BiologicalProcess	peptide cross-linking	20	2.41E-10	CSTA, IVL, SPRR1A, SPRR3, TGM1,
GO:0030216	GO_BiologicalProcess	keratinocyte differentiation	7.82609	3.61E-11	CSTA, FLG, IVL, KRT17, SPRR1A, SPRR3, TGM1, TGM3, YAP1
GO:0031424	GO_BiologicalProcess	keratinization	13.3333	3.20E-09	IVL, KRT17, SPRR1A, SPRR3, TGM1,
**Enrichment analysis of GO biological processes and KEGG pathways for top 50 DEGs**					
KEGG:03030	KEGG	DNA replication	8.33333	1.84E-04	MCM2, MCM6, RFC4
KEGG:04110	KEGG	Cell cycle	4.03226	3.89E-05	ATR, BUB1B, CDK1, MCM2, MCM6
KEGG:04115	KEGG	p53 signaling pathway	4.41176	0.001202892	ATR, BID, CDK1
GO:0006271	GO_BiologicalProcess	DNA strand elongation involved in DNA replication	10	6.75E-06	GINS1, MCM2, MCM6, RFC4

GOID	Ontology Source	GOTerm	% Associa	Term P Value	Associated Genes Found
GO:0007088	GO_BiologicalProcess	regulation of mitosis	4.38597	2.60E-05	BUB1B, CENPF, DLGAP5, RANBP1,
GO:0007091	GO_BiologicalProcess	metaphase/anaphase transition of mitotic cell cycle	7.54717	2.10E-05	BUB1B, CENPF, DLGAP5, UBE2C
GO:0007094	GO_BiologicalProcess	mitotic spindle assembly checkpoint	7.14286	2.92E-04	BUB1B, CENPF, UBE2C
GO:0022616	GO_BiologicalProcess	DNA strand elongation	9.30233	9.06E-06	GINS1, MCM2, MCM6, RFC4
GO:0030071	GO_BiologicalProcess	regulation of mitotic metaphase/anaphase transition	7.84314	1.80E-05	BUB1B, CENPF, DLGAP5, UBE2C
GO:0031577	GO_BiologicalProcess	spindle checkpoint	5.55556	6.14E-04	BUB1B, CENPF, UBE2C
GO:0032392	GO_BiologicalProcess	DNA geometric change	6	4.89E-04	MCM2, MCM6, RUVBL1
GO:0032508	GO_BiologicalProcess	DNA duplex unwinding	6.25	4.34E-04	MCM2, MCM6, RUVBL1
GO:0033627	GO_BiologicalProcess	cell adhesion mediated by integrin	6.81818	3.35E-04	EFNA1, PLAU, SNAI2
GO:0033628	GO_BiologicalProcess	regulation of cell adhesion mediated by	8.33333	1.84E-04	EFNA1, PLAU, SNAI2
GO:0034502	GO_BiologicalProcess	protein localization to chromosome	11.1111	7.67E-05	BUB1B, CDK1, CENPA
GO:0034508	GO_BiologicalProcess	centromere complex assembly	6.81818	3.35E-04	CENPA, CENPF, RUVBL1
GO:0044784	GO_BiologicalProcess	metaphase/anaphase transition of cell cycle	7.27273	2.44E-05	BUB1B, CENPF, DLGAP5, UBE2C
GO:0045839	GO_BiologicalProcess	negative regulation of mitosis	5.76923	5.49E-04	BUB1B, CENPF, UBE2C
GO:0045841	GO_BiologicalProcess	negative regulation of mitotic metaphase/anaphase transition	6.97674	3.13E-04	BUB1B, CENPF, UBE2C
GO:0051784	GO_BiologicalProcess	negative regulation of nuclear division	4.83871	9.20E-04	BUB1B, CENPF, UBE2C
GO:0071173	GO_BiologicalProcess	spindle assembly checkpoint	6.97674	3.13E-04	BUB1B, CENPF, UBE2C
GO:0071174	GO_BiologicalProcess	mitotic spindle checkpoint	6.66667	3.58E-04	BUB1B, CENPF, UBE2C
GO:0071479	GO_BiologicalProcess	cellular response to ionizing radiation	5.26316	7.19E-04	ATR, ECT2, SNAI2
GO:1902099	GO_BiologicalProcess	regulation of metaphase/anaphase transition of cell cycle	7.54717	2.10E-05	BUB1B, CENPF, DLGAP5, UBE2C
GO:1902100	GO_BiologicalProcess	negative regulation of metaphase/anaphase transition of cell cycle	6.66667	3.58E-04	BUB1B, CENPF, UBE2C

Enrichment analysis of GO biological processes and KEGG pathways for SDNs

GO:0000184	GO_BiologicalProcess	nuclear-transcribed mRNA catabolic process, nonsense-mediated decay	12.0301	3.02E-22	PPP2R1A, RBM8A, RPL10, RPL12, RPL18, RPL3, RPL7, RPLP2, RPS15A, RPS17, RPS18, RPS2, RPS28, RPS3, RPS6, RPS8
GO:0000380	GO_BiologicalProcess	alternative mRNA splicing, via spliceosome	9.09091	1.25E-04	HNRNPA1, HNRNPM, RBM8A

Table S6 Enriched biological processes and KEGG pathways of SDNs, DEPs, DEGs, and SDN-TNMs.

GOID	Ontology Source	GOTerm	% Associ	Term P Value	Associated Genes Found
GO:0000956	GO_BiologicalProcess	nuclear-transcribed mRNA catabolic process	8.0402	2.32E-19	PPP2R1A, RBM8A, RPL10, RPL12, RPL18, RPL3, RPL7, RPLP2, RPS15A, RPS17, RPS18, RPS2, RPS28, RPS3, RPS6, RPS8
GO:0001844	GO_BiologicalProcess	membrane involved in apoptotic signaling pathway	16.6667	2.48E-08	YWHAB, YWHAE, YWHAG, YWHAH, YWHAZ
GO:0002576	GO_BiologicalProcess	platelet degranulation	4.90196	1.24E-05	ACTN1, ALB, FLNA, FN1, SYK
KEGG:03010	KEGG	Ribosome	10.3704	1.48E-18	RPL10, RPL12, RPL18, RPL3, RPL7, RPLP2, RPS15A, RPS17, RPS18, RPS2, RPS28, RPS3, RPS6, RPS8
KEGG:04110	KEGG	Cell cycle	4.03226	3.18E-05	YWHAB, YWHAE, YWHAG, YWHAH,
KEGG:04114	KEGG	Oocyte meiosis	6.36364	3.21E-08	MAPK1, PPP2R1A, YWHAB, YWHAE, YWHAG, YWHAH, YWHAZ
KEGG:04390	KEGG	Hippo signaling pathway	5.19481	1.52E-08	ACTB, CTNNB1, PPP2R1A, YWHAB, YWHAE, YWHAG, YWHAH, YWHAZ
KEGG:04510	KEGG	Focal adhesion	4.36893	7.95E-09	ACTB, ACTN1, CAV1, CTNNB1, FLNA, FN1, GRB2, ITGB1, MAPK1
KEGG:04520	KEGG	Adherens junction	5.47945	6.35E-05	ACTB, ACTN1, CTNNB1, MAPK1
KEGG:04662	KEGG	B cell receptor signaling pathway	4.16667	0.001260306	GRB2, MAPK1, SYK
KEGG:04664	KEGG	Fc epsilon RI signaling pathway	4.28571	0.001161564	GRB2, MAPK1, SYK
KEGG:04917	KEGG	Prolactin signaling pathway	5.55556	6.01E-05	GRB2, MAPK1, STAT1, STAT3
KEGG:05100	KEGG	Bacterial invasion of epithelial cells	6.57895	2.90E-06	ACTB, CAV1, CTNNB1, FN1, ITGB1
KEGG:05130	KEGG	Pathogenic Escherichia coli infection	7.27273	2.07E-05	ACTB, CTNNB1, ITGB1, YWHAZ
KEGG:05131	KEGG	Shigellosis	4.91803	7.78E-04	ACTB, ITGB1, MAPK1
KEGG:05160	KEGG	Hepatitis C	4.51128	2.52E-06	EIF2S1, GRB2, MAPK1, PPP2R1A, STAT1,
KEGG:05161	KEGG	Hepatitis B	4.79452	2.25E-07	DDB1, GRB2, MAPK1, STAT1, STAT3, YWHAB, YWHAZ
KEGG:05203	KEGG	Viral carcinogenesis	5.33981	1.61E-11	SYK, YWHAB, YWHAE, YWHAG, YWHAH, YWHAZ
KEGG:05205	KEGG	Proteoglycans in cancer	4.44444	8.99E-10	ACTB, CAV1, CTNNB1, FLNA, FN1, GRB2, ITGB1, MAPK1, RPS6, STAT3
KEGG:05212	KEGG	Pancreatic cancer	4.54545	9.79E-04	MAPK1, STAT1, STAT3
KEGG:05213	KEGG	Endometrial cancer	5.76923	4.87E-04	CTNNB1, GRB2, MAPK1
KEGG:05221	KEGG	Acute myeloid leukemia	5.26316	6.38E-04	GRB2, MAPK1, STAT3

Table S6 Enriched biological processes and KEGG pathways of SDNs, DEPs, DEGs, and SDN-TNMs.

GOID	Ontology Source	GOTerm	% Associa	Term P Value	Associated Genes Found
KEGG:05412	KEGG	Arrhythmogenic right ventricular cardiomyopathy (ARVC)	5.40541	6.70E-05	ACTB, ACTN1, CTNNB1, ITGB1
GO:0006364	GO_BiologicalProcess	rRNA processing	4.20168	2.61E-05	RPL7, RPS17, RPS28, RPS6, RPS8
GO:0006401	GO_BiologicalProcess	RNA catabolic process	6.58436	5.86E-18	PPP2R1A, RBM8A, RPL10, RPL12, RPL18, RPL3, RPL7, RPLP2, RPS15A, RPS17, RPS18, RPS2, RPS28, RPS3, RPS6, RPS8
GO:0006402	GO_BiologicalProcess	mRNA catabolic process	7.47664	7.55E-19	PPP2R1A, RBM8A, RPL10, RPL12, RPL18, RPL3, RPL7, RPLP2, RPS15A, RPS17, RPS18, RPS2, RPS28, RPS3, RPS6, RPS8
GO:0006413	GO_BiologicalProcess	translational initiation	9.35673	1.95E-20	EIF2S1, KHDRBS1, RPL10, RPL12, RPL18, RPL3, RPL7, RPLP2, RPS15A, RPS17, RPS18, RPS2, RPS28, RPS3, RPS6, RPS8
GO:0006414	GO_BiologicalProcess	translational elongation	12.3967	4.54E-21	EEF1A1, RPL10, RPL12, RPL18, RPL3, RPL7, RPLP2, RPS15A, RPS17, RPS18, RPS2, RPS28, RPS3, RPS6, RPS8
GO:0006415	GO_BiologicalProcess	translational termination	13.8614	2.12E-20	RPL10, RPL12, RPL18, RPL3, RPL7, RPLP2, RPS15A, RPS17, RPS18, RPS2, RPS28, RPS3, RPS6, RPS8
GO:0006605	GO_BiologicalProcess	protein targeting	4.18182	1.44E-21	RPL12, RPL18, RPL3, RPL7, RPLP2, RPS15A, RPS17, RPS18, RPS2, RPS28, RPS3, RPS6, RPS8, STAT3, YWHAB, YWHAE, YWHAG, YWHAZ
GO:0006612	GO_BiologicalProcess	protein targeting to membrane	7.97872	4.13E-18	PSMD2, RPL10, RPL12, RPL18, RPL3, RPL7, RPLP2, RPS15A, RPS17, RPS18, RPS2, RPS28, RPS3, RPS6, RPS8
GO:0006613	GO_BiologicalProcess	cotranslational protein targeting to membrane	12.5	3.99E-21	PSMD2, RPL10, RPL12, RPL18, RPL3, RPL7, RPLP2, RPS15A, RPS17, RPS18, RPS2, RPS28, RPS3, RPS6, RPS8
GO:0006614	GO_BiologicalProcess	SRP-dependent cotranslational protein targeting to membrane	12.7119	3.06E-21	PSMD2, RPL10, RPL12, RPL18, RPL3, RPL7, RPLP2, RPS15A, RPS17, RPS18, RPS2, RPS28, RPS3, RPS6, RPS8
GO:0007006	GO_BiologicalProcess	mitochondrial membrane organization	5.61798	6.34E-06	YWHAB, YWHAE, YWHAG, YWHAH,
GO:0010821	GO_BiologicalProcess	regulation of mitochondrion organization	5.10204	1.02E-05	YWHAB, YWHAE, YWHAG, YWHAH,

Table S6 Enriched biological processes and KEGG pathways of SDNs, DEPs, DEGs, and SDN-TNMs.

GOID	Ontology Source	GOTerm	% Associ	Term P Value	Associated Genes Found
GO:0010822	GO_BiologicalProcess	positive regulation of mitochondrion organization	8.33333	8.90E-07	YWHAB, YWHAE, YWHAG, YWHAH, YWHAZ
GO:0016072	GO_BiologicalProcess	rRNA metabolic process	4	3.31E-05	RPL7, RPS17, RPS28, RPS6, RPS8
GO:0019058	GO_BiologicalProcess	viral life cycle	5.81818	4.24E-17	KPNB1, RPL10, RPL12, RPL18, RPL3, RPL7, RPLP2, RPS15A, RPS17, RPS18, RPS2, RPS28, RPS3, RPS6, RPS8, UBC
GO:0019080	GO_BiologicalProcess	viral gene expression	9.55414	2.61E-19	RPL10, RPL12, RPL18, RPL3, RPL7, RPLP2, RPS15A, RPS17, RPS18, RPS2, RPS28, RPS3, RPS6, RPS8, UBC
GO:0019083	GO_BiologicalProcess	viral transcription	9.45946	5.55E-18	RPL10, RPL12, RPL18, RPL3, RPL7, RPLP2, RPS15A, RPS17, RPS18, RPS2, RPS28, RPS3, RPS6, RPS8
GO:0021539	GO_BiologicalProcess	subthalamus development	5	7.41E-04	ACTB, YWHAE, YWHAH
GO:0021762	GO_BiologicalProcess	substantia nigra development	5.17241	6.71E-04	ACTB, YWHAE, YWHAH
GO:0032984	GO_BiologicalProcess	macromolecular complex disassembly	6.51163	1.11E-15	RPL10, RPL12, RPL18, RPL3, RPL7, RPLP2, RPS15A, RPS17, RPS18, RPS2, RPS28, RPS3, RPS6, RPS8
GO:0035794	GO_BiologicalProcess	positive regulation of mitochondrial membrane permeability	8.92857	6.28E-07	YWHAB, YWHAE, YWHAG, YWHAH, YWHAZ
GO:0042254	GO_BiologicalProcess	ribosome biogenesis	4.14201	6.09E-07	EIF2S1, RPL7, RPS17, RPS18, RPS28,
GO:0042274	GO_BiologicalProcess	ribosomal small subunit biogenesis	16.6667	6.89E-07	RPS17, RPS28, RPS6, RPS8
GO:0043241	GO_BiologicalProcess	protein complex disassembly	6.73077	6.97E-16	RPL10, RPL12, RPL18, RPL3, RPL7, RPLP2, RPS15A, RPS17, RPS18, RPS2, RPS28, RPS3, RPS6, RPS8
GO:0043624	GO_BiologicalProcess	cellular protein complex disassembly	7.52688	1.44E-16	RPL10, RPL12, RPL18, RPL3, RPL7, RPLP2, RPS15A, RPS17, RPS18, RPS2, RPS28, RPS3, RPS6, RPS8
GO:0045047	GO_BiologicalProcess	protein targeting to ER	12.5	3.99E-21	PSMD2, RPL10, RPL12, RPL18, RPL3, RPL7, RPLP2, RPS15A, RPS17, RPS18, RPS2, RPS28, RPS3, RPS6, RPS8
GO:0046902	GO_BiologicalProcess	regulation of mitochondrial membrane permeability	7.57576	1.44E-06	YWHAB, YWHAE, YWHAG, YWHAH, YWHAZ
GO:0051204	GO_BiologicalProcess	protein insertion into mitochondrial	16.129	2.95E-08	YWHAB, YWHAE, YWHAG, YWHAH,
GO:0051205	GO_BiologicalProcess	protein insertion into membrane	12.1951	1.27E-07	YWHAB, YWHAE, YWHAG, YWHAH,

Table S6 Enriched biological processes and KEGG pathways of SDNs, DEPs, DEGs, and SDN-TNMs.

GOID	Ontology Source	GOTerm	% Associ:	Term P Value	Associated Genes Found
GO:0051651	GO_BiologicalProcess	maintenance of location in cell	4.23729	2.51E-05	ALB, CAV1, CTNNB1, FLNA, YWHAB
GO:0051668	GO_BiologicalProcess	localization within membrane	9.61539	4.31E-07	YWHAB, YWHAE, YWHAG, YWHAH,
GO:0060396	GO_BiologicalProcess	growth hormone receptor signaling pathway	9.375	1.14E-04	MAPK1, STAT1, STAT3
GO:0060397	GO_BiologicalProcess	JAK-STAT cascade involved in growth hormone signaling pathway	12.5	4.73E-05	MAPK1, STAT1, STAT3
GO:0060416	GO_BiologicalProcess	response to growth hormone	7.14286	2.58E-04	MAPK1, STAT1, STAT3
GO:0070585	GO_BiologicalProcess	protein localization to mitochondrion	5.55556	6.70E-06	YWHAB, YWHAE, YWHAG, YWHAH,
GO:0070972	GO_BiologicalProcess	protein localization to endoplasmic reticulum	11.0294	2.83E-20	PSMD2, RPL10, RPL12, RPL18, RPL3, RPL7, RPLP2, RPS15A, RPS17, RPS18, RPS2, RPS28, RPS3, RPS6, RPS8
GO:0071378	GO_BiologicalProcess	cellular response to growth hormone	8.57143	1.49E-04	MAPK1, STAT1, STAT3
GO:0072594	GO_BiologicalProcess	establishment of protein localization to organelle	4.8583	3.91E-24	RPL12, RPL18, RPL3, RPL7, RPLP2, RPS15A, RPS17, RPS18, RPS2, RPS28, RPS3, RPS6, RPS8, STAT3, YWHAB, YWHAE, YWHAG, YWHAH, YWHAZ
GO:0072599	GO_BiologicalProcess	establishment of protein localization to endoplasmic reticulum	12.3967	4.54E-21	PSMD2, RPL10, RPL12, RPL18, RPL3, RPL7, RPLP2, RPS15A, RPS17, RPS18, RPS2, RPS28, RPS3, RPS6, RPS8
GO:0072655	GO_BiologicalProcess	establishment of protein localization to mitochondrion	5.61798	6.34E-06	YWHAB, YWHAE, YWHAG, YWHAH, YWHAZ
GO:0072657	GO_BiologicalProcess	protein localization to membrane	5.69106	2.31E-22	RPL3, RPL7, RPLP2, RPS15A, RPS17, RPS18, RPS2, RPS28, RPS3, RPS6, RPS8, YWHAB, YWHAE, YWHAG, YWHAH, YWHAZ
GO:0090150	GO_BiologicalProcess	establishment of protein localization to membrane	6.53595	1.85E-22	PSMD2, RPL10, RPL12, RPL18, RPL3, RPL7, RPLP2, RPS15A, RPS17, RPS18, RPS2, RPS28, RPS3, RPS6, RPS8, YWHAB, YWHAE, YWHAG, YWHAH, YWHAZ
GO:0097345	GO_BiologicalProcess	mitochondrial outer membrane	9.43396	4.75E-07	YWHAB, YWHAE, YWHAG, YWHAH,
GO:1900739	GO_BiologicalProcess	regulation of protein insertion into mitochondrial membrane involved in apoptotic signaling pathway	17.8571	1.72E-08	YWHAB, YWHAE, YWHAG, YWHAH, YWHAZ

Table S6 Enriched biological processes and KEGG pathways of SDNs, DEPs, DEGs, and SDN-TNMs.

GOID	Ontology Source	GOTerm	% Associ	Term P Value	Associated Genes Found
GO:1900740	GO_BiologicalProcess	positive regulation of protein insertion into mitochondrial membrane involved in apoptotic signaling pathway	17.8571	1.72E-08	YWHAB, YWHAE, YWHAG, YWHAH, YWHAZ
GO:1901028	GO_BiologicalProcess	regulation of mitochondrial outer membrane permeabilization involved in apoptotic signaling pathway	11.9048	1.44E-07	YWHAB, YWHAE, YWHAG, YWHAH, YWHAZ
GO:1902108	GO_BiologicalProcess	regulation of mitochondrial membrane permeability involved in apoptotic process	8.77193	6.87E-07	YWHAB, YWHAE, YWHAG, YWHAH, YWHAZ
GO:1902110	GO_BiologicalProcess	membrane permeability involved in apoptotic process	9.09091	5.73E-07	YWHAB, YWHAE, YWHAG, YWHAH, YWHAZ
GO:1902580	GO_BiologicalProcess	single-organism cellular localization	4.86111	6.23E-21	RPL3, RPL7, RPLP2, RPS15A, RPS17, RPS18, RPS2, RPS28, RPS3, RPS6, RPS8, YWHAB, YWHAE, YWHAG, YWHAH, YWHAZ
GO:1902686	GO_BiologicalProcess	permeabilization involved in programmed cell death	9.09091	5.73E-07	YWHAB, YWHAE, YWHAG, YWHAH, YWHAZ

Table S6 Enriched biological processes and KEGG pathways of SDNs, DEPs, DEGs, and SDN-TNMs.

Supplemental file 1 SAS code for factorial analysis

```
data escc;
input ID$ x1-x7;
cards;
.../*Paste data in the excel file named: Normalized topological indexes*/
;

proc factor data=escc priors=smc    /*?Perform factorial analysis*/
rotate=promax reorde /*Coordinate rotation to easier explanation*/
plots=(scree initloadings preloadings loadings) /*Plot figures*/
nfactors=2 /*Keep the first two factors*/
score out=esccresult;      /*output factor scores and save the factorial analysis results*/
var x1--x7;
run;


proc sort data=esccresult;/*Rank nodes by factor1*/
by factor1;
run;


proc print data=esccresult; /*Print results*/
run;
```
